# scPharm: Identifying Pharmacological Subpopulations of Single Cells for Precision Medicine in Cancers

**DOI:** 10.1002/advs.202412419

**Published:** 2024-11-19

**Authors:** Peng Tian, Jie Zheng, Keying Qiao, Yuxiao Fan, Yue Xu, Tao Wu, Shuting Chen, Yinuo Zhang, Bingyue Zhang, Chiara Ambrogio, Haiyun Wang

**Affiliations:** ^1^ Research Center for Translational Medicine Shanghai East Hospital School of Life Sciences and Technology Tongji University Shanghai 200092 China; ^2^ Department of Molecular Biotechnology and Health Sciences Molecular Biotechnology Center University of Torino Torino 10126 Italy

**Keywords:** intratumour heterogeneity, pharmacological subpopulations, precision medicine, scPharm

## Abstract

Intratumour heterogeneity significantly hinders the efficacy of anticancer therapies. Compared with drug perturbation experiments, which yield pharmacological data at the bulk cell level, single‐cell RNA sequencing (scRNA‐seq) technology provides a means to capture molecular heterogeneity at single‐cell resolution. Here, scPharm is introduced, a computational framework that integrates pharmacological profiles with scRNA‐seq data to identify pharmacological subpopulations of cells within a tumour and prioritize tailored drugs. scPharm uses the normalized enrichment scores (NESs) determined from gene set enrichment analysis to assess the distribution of cell identity genes in drug response‐determined gene lists. Based on the strong correlation between the NES and drug response at single‐cell resolution, scPharm successfully identified the sensitive subpopulations in ER‐positive and HER2‐positive human breast cancer tissues, revealed dynamic changes in the resistant subpopulation of human PC9 cells treated with erlotinib, and expanded its ability to a mouse model. Its superior performance and computational efficiency are confirmed through comparative evaluations with other single‐cell prediction tools. Additionally, scPharm predicted combination drug strategies by gauging compensation or booster effects between drugs and evaluated drug toxicity in healthy cells in the tumour microenvironment. Overall, scPharm provides a novel approach for precision medicine in cancers by revealing therapeutic heterogeneity at single‐cell resolution.

## Graphical abstract

The study presents scPharm, a computational framework that integrates pharmacological data with scRNA‐seq data to identify pharmacological subpopulations within a tumour. By analyzing normalized enrichment scores (NES) of single cells in the context of drug response, scPharm effectively pinpoints sensitive and resistant subpopulations in cancers, predicts combination therapies, and assesses drug toxicity, offering a promising approach to precision oncology.

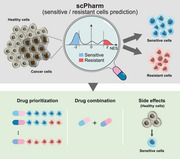

## 1 Introduction

The intrinsic heterogeneity of individual tumours poses a significant challenge in cancer treatment, often leading to the failure of anticancer therapies and patient death. Recent advances in single‐cell sequencing technology have created opportunities for investigating molecular heterogeneity at the single‐cell level and have shown promise in precision cancer therapy. In recent years, technologies such as Perturb‐seq,^[^ ^1^ ^]^ expanded CRISPR‐compatible cellular indexing of transcriptomes and epitopes by sequencing (ECCITE‐seq)^[^ ^2^ ^]^ and sci‐Plex^[^ ^3^ ^]^ have integrated single‐cell sequencing with the compound‐ or clustered regularly interspaced short palindromic repeats (CRISPR)‐mediated gene perturbation experiments, allowing the assessment of single‐cell perturbation responses at the transcriptome or surface protein level. Despite their potential, these biotechnological platforms are still in their infancy.

Notably, the application of perturbation‐based screening of bulk cells of cancer cell lines in vivo over the past decade has proven valuable in identifying cancer cell targets. Moreover, this screening method has produced large‐scale genetic characterizations of cancer cell lines, as well as their phenotypes, through perturbation experiments. For example, the pharmacogenomic projects Cancer Cell Line Encyclopedia (CCLE)^[^ ^4^ ^]^ and Genomics of Drug Sensitivity in Cancer (GDSC)^[^ ^5^ ^]^ performed sequencing of over 1000 tumour cell lines and sensitivity analyses of hundreds of drugs. The Cancer Therapeutics Response Portal (CTRP)^[^ ^6^ ^]^ has tested the drug responses of almost 900 cancer cell lines to nearly 500 drugs. In addition, projects integrating CRISPR screening platforms with the concept of joint lethality, including Project Score,^[^ ^7^ ^]^ Project Achilles,^[^ ^8^ ^]^ and Project DRIVE,^[^ ^9^ ^]^ identified genetic perturbations required for cell fitness from a molecular perspective. Given these advancements, the potential exists to develop a computational pipeline that integrates single‐cell transcriptomic profiles with pharmacogenomic profiles to predict the pharmacological phenotype of single cells, addressing the limitations of current biotechnology platforms.

While several methods have been developed to predict drug responses at the single‐cell level, these approaches can be broadly categorized into machine learning‐based strategies and alternative computational methods. Machine learning approaches, such as SCAD,^[^ ^10^ ^]^ scRank,^[^ ^11^ ^]^ scDEAL,^[^ ^12^ ^]^ and CaDRReS‐Sc,^[^ ^13^ ^]^ often leverage bulk and single‐cell data to predict drug sensitivity and identify resistant subpopulations. These methods rely on transfer learning, feature extraction, and deep learning to capture the heterogeneity within tumours. On the other hand, information–theoretic analyses, such as CSSS,^[^ ^14^ ^]^ and hybrid methods, such as Scissor,^[^ ^15^ ^]^ provide complementary perspectives to machine learning‐driven strategies. Despite these advancements, challenges remain in scaling these models and improving their interpretability for clinical applications. Furthermore, publicly available pharmacogenomic datasets are still limited in size compared with the complexity of the model. Another limitation is the reliance on binarized drug response labels, such as “sensitive” versus “resistant”, which often depend on binarization methods lacking clear biological interpretation.^[^ ^16^ ^]^ Additionally, most existing methods construct models solely to predict whether single cells are sensitive or resistant to drugs, overlooking the broader clinical context of drug efficacy and side effects.

Here, we propose scPharm, a computational framework applicable to real‐world single‐cell RNA sequencing (scRNA‐seq) data of tumour tissues, with the aim of addressing these challenges. In such real‐world scenarios, tumour tissues often consist of a mixture of various cell types within the tumour microenvironment rather than comprising solely pure cancer cells. We hypothesize that if a single cell is sensitive to a drug, the highly expressed genes in this cell, termed identity genes (Cell‐ID), would significantly overlap with genes associated with increased drug sensitivity. Conversely, if a single cell is resistant, the highly expressed genes would overlap with those correlated with increased drug resistance. Our approach employed gene set enrichment analysis (GSEA)^[^ ^17^ ^]^ as a paradigm, dynamically calculating the Cell‐ID gene set based on scRNA‐seq data. Unlike regular GSEA, which uses a stable set of a priori defined genes, scPharm utilizes the dynamically calculated identity genes of each individual cell. Accordingly, a list of genes is ranked according to the drug response, which is generated by the correlation of gene expression with drug response based on bulk data. The gene list per drug within the same cancer type is stable and applicable to all single cells.

In contrast to machine learning models that transfer drug response phenotypes from bulk data to single cells based on similarities in expression profiles, scPharm utilizes the normalized enrichment score (NES) derived from GSEA to identify statistically significant sensitive and resistant single cells. This statistical methodology overcomes challenges related to small sample sizes, binarization in machine learning models, and the integration of expression similarity across modalities. The performance of scPharm was comprehensively assessed across various datasets, including scRNA‐seq data from ER‐positive and HER2‐positive breast cancer tissues, PC9 cells treated with erlotinib at different time points, and the MMTV‐PyMT mouse mammary tumour model. Additionally, a rigorous comparative analysis was conducted to assess the predictive performance of scPharm against other single‐cell prediction tools, including scDEAL,^[^ ^12^ ^]^ CaDRReS‐Sc,^[^ ^13^ ^]^ Scissor^[^ ^15^ ^]^ and SeuratCCA.^[^ ^18^ ^]^

Notably, the functions of scPharm extend beyond prediction, providing valuable insights into the prioritization of tailored drugs. Its distinctive feature also lies in the assessment of combination strategies, gauging compensation effects or booster effects between two drugs through the set covering method. Moreover, scPharm evaluates drug side effects by leveraging scRNA‐seq data from healthy human tissues, enabling the assessment of drug toxicity in healthy cells within the tumour microenvironment.

## 2 Results

### 2.1 Overview of scPharm

**Figure 1:**
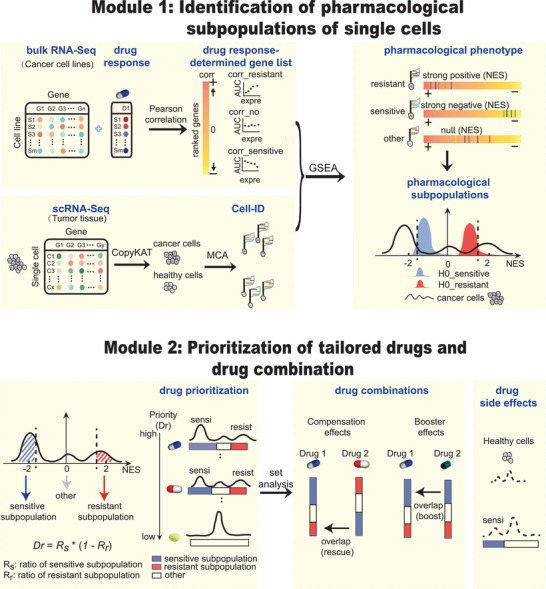
Overview of the computational framework scPharm. scPharm consists of two modules. Module 1 uses pharmacogenomic data and scRNA‐seq data to identify statistically significant sensitive and resistant single‐cell subpopulations. GSEA, as a paradigm, determines whether a single cell is sensitive to a drug. Module 2 prioritizes tailored drugs by *Dr* values and develops drug combination strategies by evaluating whether two drugs exhibit compensation effects or booster effects.

scPharm is a two‐module computational framework designed to integrate large‐scale pharmacogenomic profiles with scRNA‐seq data (**Figure** **1**, see the Experimental Section). scPharm is applied to scRNA‐seq data obtained from real‐world tumour tissue samples involving a mixture of various cell types within the tumour microenvironment. scPharm first employs CopyKAT^[^ ^19^ ^]^ to differentiate tumour cells from healthy cells. **Module 1** identifies pharmacological subpopulations of single tumour cells. Two inputs are used: 1) the drug response‐determined gene list calculated from bulk RNA‐seq and corresponding drug response data from the GDSC dataset, where genes at the top correlate with drug resistance and those at the bottom correlate with drug sensitivity, and 2) the Cell‐ID gene set of single cells extracted using multiple correspondence analysis (MCA).^[^ ^20^ ^]^ The module uses the NES obtained from GSEA to assess the distribution of the Cell‐ID within the ranked gene list and to predict the drug response of a single cell. Performing this analysis on all cells in a tumour generates a distribution of NESs that can be used to infer pharmacological subpopulations with a specific response to a drug. **Module 2** evaluates drug suitability using the *Dr* values, which is calculated from the proportions of sensitive and resistant subpopulations and prioritizes the tailored drugs. It further explores potential combinatorial therapies, gauging compensation effects or booster effects between two drugs, and evaluates potential drug toxicity to healthy cells within the tumour microenvironment.

We modeled the statistical tests in Module 1 using scRNA‐seq data from healthy human tissues and pooled drugs to generate null distribution (*H0*) of NESs. We constructed the null distribution using healthy human tissues because this approach better represents the differences between tumour and normal cells, which is crucial for identifying tumour‐specific vulnerabilities. Our objective was to identify drugs that could selectively target tumour cells while sparing healthy cells. By using healthy tissue to construct the null distribution, we created a more stringent baseline that distinguishes drug effects on tumour cells from those on normal cells, which is biologically meaningful in the context of therapeutic targeting. We investigated three types of healthy human tissues, including lung, breast and skin tissues. The three tissue types exhibited very similar distribution characteristics, featuring two peaks, one with a median of ≈−1 and the other with a median of ≈+1 (Figure 1; Figure S1, Supporting Information). Therefore, we pooled the three types of healthy tissues and all drugs to generate a unique null distribution. Gaussian mixture model decomposition (see the Experimental Section)^[^ ^21^ ^]^ was then performed to estimate the parameters of two separate *H0* distributions accurately for sensitive cells (*H0*_sensitive) and resistant cells (*H0*_resistant).

In our model, two primary parameters were optimized: ^(1)^ the size of the Cell‐ID and ^(2)^ the threshold of the NES. The optimal Cell‐ID size was determined by performing a quantitative gradient experiment to evaluate several different values: 100, 150, 200, 250, and 300. Across these settings, the predictive performance of scPharm remained stable, with only minor fluctuations observed in the results. Since 200 is the default value for the number of features in the MCA method and given that our experiment confirmed the stability at this value, we selected 200 as the optimal Cell‐ID size (Figure S2A, Supporting Information). For the NES threshold, we used a unified threshold based on the mean ± one standard deviation for *H0*_sensitive and *H0*_resistant individually, which are more stringent than the gene‐wise perturbation thresholds, calculated by shuffling drug response‐determined gene list. This approach results in more accurate drug prioritizations, as it minimizes the risk of false positives (Figure S2B, Supporting Information). Using the optimized thresholds, scPharm categorizes single tumour cells into sensitive or resistant subpopulations for a given drug.

### 2.2 Rationale of scPharm

scPharm allows users to predict the drug response using NESs. We hypothesize that if a single cell is sensitive to a drug, its Cell‐ID would significantly overlap with genes whose high expression correlates with increased drug sensitivity (sensitive markers); conversely, if a single cell is resistant to a drug, its Cell‐ID would significantly overlap with those genes correlated with increased drug resistance (resistant markers). GSEA was employed to address this issue. Our study created a drug response‐determined gene list using cell lines from the same cancer type to mitigate the influence of tissue‐specific expression on the predictions.

**Figure 2:**
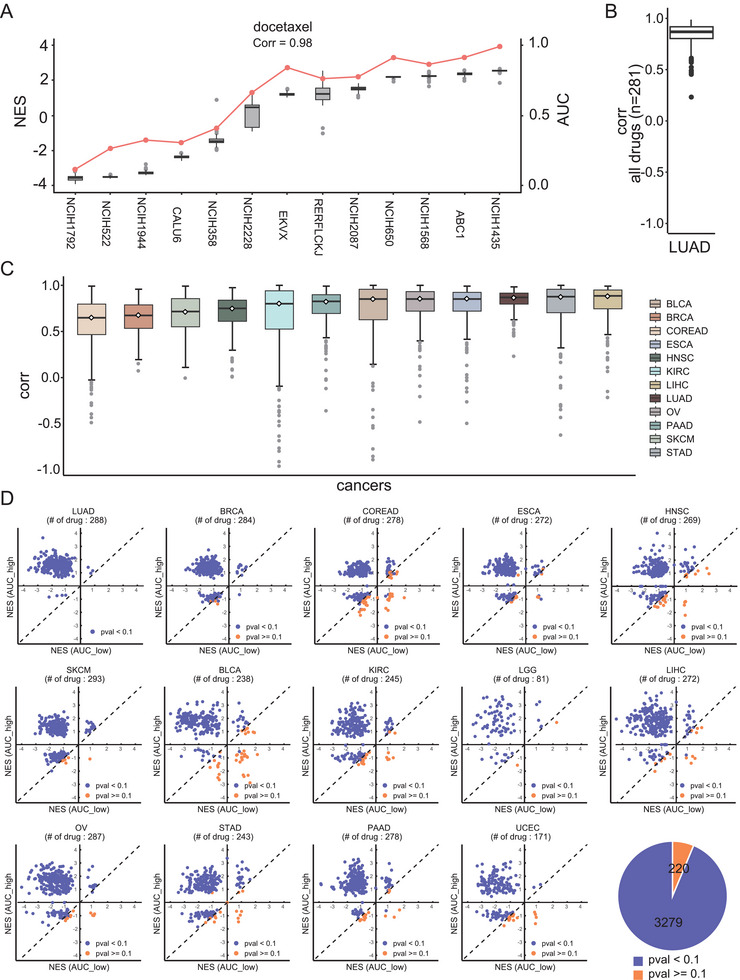
Correlation between the NES and drug response. A) The red line depicts the drug responses of 13 lung adenocarcinoma (LUAD) cell lines to docetaxel, which were measured using an AUC. The boxplot depicts the NESs for single cells in the corresponding LUAD cell lines. The Pearson correlation coefficients (Corr) between the AUCs and median NESs of single cells are shown. B) Boxplot of Pearson correlation coefficients between the AUCs and median NESs for all drugs in LUAD cell lines. C) Boxplot of Pearson correlation coefficients across the different cancers. D) Scatter plot showing the NESs in the AUC_high group versus the AUC_low group. The cell lines were divided into AUC_high and AUC_low groups based on the median AUC for each drug. A one‐sided Mann‒Whitney test was conducted to compare the NESs between these two groups. Each dot represents a drug, with the horizontal coordinate showing the median NES of cells from the AUC_high group and the vertical coordinate showing the median NES of cells from the AUC_low group. Dots located in the upper left of the diagonal indicate that the median NES of cells from the AUC_high group is greater than that of the AUC_low group, with blue dots corresponding to drugs with *p* < 0.1. Conversely, dots in the lower right of the diagonal indicate that the median NES of cells from the AUC_high group is lower than that from the AUC_low group, with orange dots representing drugs with *p* ≥ 0.1. The pie chart depicts the number of drugs with significant *p* values for the correlations between the AUCs and NESs across cancers.

We first observed the correlation between the NES and drug response at the single‐cell level to test these hypotheses. The scRNA‐seq data of human cancer cell lines and drug response data, which were quantified with the area under the dose–response curve (AUC), from the cell population of the same cancer cell lines were collected (see the Experimental Section).^[^ ^5^ ^]^ We employed GSEA to compute the NESs of single cells and then calculated the Pearson correlation coefficient between the NES and AUC. Our analysis included 281 drugs and ≈40 lung adenocarcinoma (LUAD) cell lines. Remarkably, we observed a very strong correlation (*R* = 0.98) for drugs such as docetaxel (**Figure** **2**A), for which 13 LUAD cell lines, with each cell line including 252 single cells, were investigated. We used the median NESs of these single cells as the summarized NES for the corresponding cell line. This robust correlation extended across 281 drugs, with a median correlation of ≈0.8 (Figure 2B), and 12 cancer types (Figure 2C).

After dividing single cells into AUC_high and AUC_low groups, further analysis revealed significantly higher NESs in the AUC_high group for most drugs (Figure 2D). These analyses using different statistical approaches provide strong evidence that the NES is robustly and strongly correlated with the drug response, demonstrating its predictive potential at the single‐cell level.

### 2.3 Identification of a Sensitive Subpopulation in HER2‐Positive Breast Cancer

To assess whether scPharm can accurately identify a sensitive subpopulation from single‐cell data, we analysed scRNA‐seq data from six HER2‐positive human breast cancer tissue samples, namely, MH0176, MH0031, MH0069, PM0337, AH0308 and MH0161. After scRNA‐seq data processing, 44761 cells were retained, with an average of 7460 cells per sample and 1356 genes per cell. These HER2‐positive samples are known to be sensitive to HER2 inhibitors.^[^ ^22^ ^]^ This evaluation specifically focused on three types of HER2 inhibitors: afatinib, sapitinib and lapatinib. We distinguished 10124 tumour cells and 34637 healthy cells from the total population of 44761 cells using CopyKAT and extracted 200 identity genes per cell using MCA.

**Figure 3:**
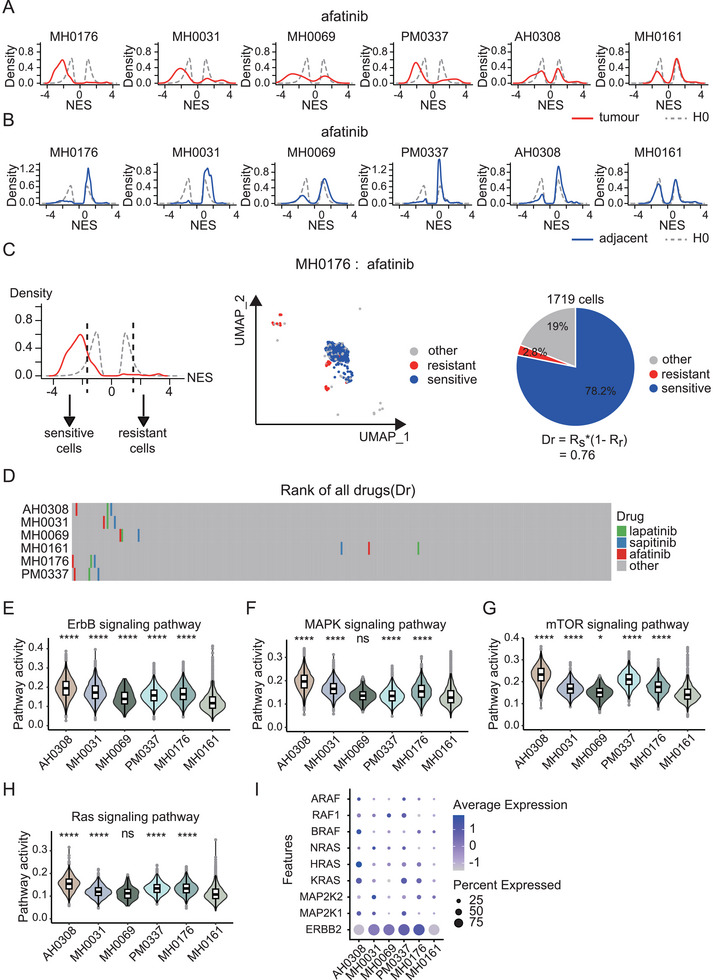
Application of scPharm in HER2‐positive breast cancer. A) Density plot depicting the NESs of single cells from six HER2‐positive breast cancer tissues (red curves) and healthy human tissues (grey curves) for afatinib. B) Density plot depicting the NESs of single cells from tumour‐adjacent tissues and healthy human tissues for afatinib. C) Plot illustrating sample MH0176 in the context of afatinib. D) Ranks of three HER2 inhibitors in HER2‐positive samples. E) Comparison of ErbB signaling pathway activity among all HER2‐positive samples. F) Comparison of MAPK signaling pathway activity among all HER2‐positive samples. G) Comparison of mTOR signaling pathway activity among all HER2‐positive samples. H) Comparison of Ras signaling pathway activity among all HER2‐positive samples. I) Expression of ERBB2 (HER2) and key genes in the ErbB signaling pathway. The Mann‐Whitney test was employed for comparisons between sample MH0161 and other samples, with the symbols “ns”, “*”, “**”, “***” and “****” representing significance levels of 0.05 ≤ *p* < 1, 0.01 ≤ *p* < 0.05, 0.001 ≤ *p* < 0.01, *p* < 0.001, and *p* < 0.0001, respectively.

For afatinib, the NES distribution curves of single tumour cells exhibited one large negative peak and several smaller peaks, typically in MH0176, MH0031, MH0069, and PM0337 (**Figure** **3**A). Interestingly, for all the samples except MH0161, the negative peak significantly shifted to the left compared to *H0*_sensitive. This result indicates the presence of a sensitive subpopulation within the tumour cells. Conversely, this phenomenon was not observed in adjacent normal cells from the same samples (Figure 3B). Additionally, similar distribution curves were also observed for other HER2‐targeted drugs, such as sapitinib and lapatinib (Figure S3, Supporting Information).

Based on the null distribution of the NES, scPharm utilized cut‐off values of −1.75 and 1.52 to determine whether the NES for a single cell significantly differed from *H0*. Then, both sensitive and resistant cell subpopulations were identified by analysing all cells in a tumour. For example, for afatinib in sample MH0176 (Figure 3C), the sensitive subpopulation included 1344 cells identified using a cut‐off of −1.75, and the resistant subpopulation included 49 cells identified with a cut‐off of 1.52. Based on the sizes of the two subpopulations, the drug score, *Dr*, for afatinib was 0.76. All 295 drugs were ranked based on their corresponding *Dr* values, as depicted in Figure 3D; the three types of HER2 inhibitors are shown in green, blue and red, with grey indicating other drugs. The results revealed that among five of the six HER2‐positive samples, HER2 inhibitors consistently achieved high scores and ranked at the top of all drugs (Figure 3D). The details of the top 30 drugs are shown in Figure S4 (Supporting Information). Notably, drugs that target EGFR, such as gefitinib, erlotinib, and osimertinib, were also highly effective, suggesting a potential role for EGFR‐targeted drugs in HER2‐positive patients.^[^ ^23^ ^]^ Additionally, the results highlight the intersample heterogeneity between different HER2‐positive breast cancer tissues. While the majority of samples are sensitive to HER2 inhibitors, the profile of recommended drugs varies significantly across different samples. This variation underscores the existence of distinct subpopulations within each tumour. The observed heterogeneity suggests that although these HER2‐positive tissues may initially be sensitive to HER2 inhibitors, the presence of distinct cellular subpopulations could lead to the development of various resistance mechanisms over time. These differences in drug sensitivity may reflect underlying genetic, epigenetic, or microenvironmental factors that drive intertumoral heterogeneity.

Notably, for sample MH0161, the three HER2 inhibitors were not highly ranked, as we expected. To elucidate the potential mechanism leading to this difference, we investigated the activity of pathways associated with HER2 inhibitors, as well as the expression of key genes in these pathways. Pathway activity was measured by calculating the average expression of genes in the pathway. The analyses revealed that the activity of the ErbB signaling pathway, a target of HER2 inhibitors, was significantly lower in MH0161 than in the other five samples (Figure 3E). Its downstream pathways, such as MAPK signaling, mTOR signaling, and Ras signaling, also exhibited decreased activity in MH0161 (Figures 3F–H). Moreover, key genes, such as *ERBB2*, *KRAS*, and *MAP2K1*, were expressed at extremely low levels in MH0161 (Figure 3I). These findings may explain the discrepancy in the rank of HER2 inhibitors in MH0161 cells.^[^ ^24^ ^]^

### 2.4 Identification of a Sensitive Subpopulation in ER‐Positive Breast Cancer

We further evaluated the performance of scPharm on the scRNA‐seq data from 13 ER‐positive breast cancer tissue samples (Figures S5–S7, Supporting Information). The analyses of the scRNA‐seq data revealed a total of 72309 cells, with an average of 5562 cells per sample and 1051 genes per cell. These ER‐positive samples are known to be sensitive to ER inhibitors.^[^ ^25^ ^]^ This evaluation focused on two types of ER inhibitors, fulvestrant and GDC0810. For fulvestrant, the GDSC dataset contained two drug response assays, corresponding to the different maximum screening concentrations. Here, we refer to them as fulvestrant_low and fulvestrant_high according to the maximum concentrations of 1 and 10 µm, respectively. We distinguished 26059 tumour cells and 46250 healthy cells from all 72309 cells and extracted 200 identity genes per cell.

For GDC0810, we observed a left‐shifted negative peak in single tumour cells across ER‐positive samples, but not in adjacent healthy cells (13 samples are shown in Figure S5A,B, Supporting Information). This result indicated the significant presence of a subpopulation sensitive to GDC0810. These characteristics extended to fulvestrant curves (Figure S6, Supporting Information). We ranked and highlighted the ER inhibitors based on the *Dr* values of 295 drugs (Figure S5C, Supporting Information). Except for MH0042, ER inhibitors were consistently highly ranked in most of the samples (Figure S5C, Supporting Information). The top 30 drugs recommended by scPharm for the four samples are shown in detail in Figure S5D (Supporting Information).

Interestingly, for the two maximum screening concentrations of fulvestrant, scPharm ranked fulvestrant_high ahead of fulvestrant_low (Figure S5C, Supporting Information). For fulvestrant_low at a maximum concentration of 1 µm, the drug response values of numerous cell lines were beyond the tested range and could not be accurately estimated. The replicated assay using the increased maximum concentration of 10 µm covered the drug response concentration for most cells to accurately estimate the drug response. Accordingly, the drug response‐determined gene list used for GSEA was more accurate for fulvestrant_high than for fulvestrant_low. This situation led to an improved ranking of fulvestrant_high in ER‐positive breast cancer samples (Figures S5C and S7A, Supporting Information).

Further analyses revealed that the subpopulation sensitive to fulvestrant_high was significantly larger than that sensitive to fulvestrant_low (Figure S7B–G, Supporting Information). Based on the changes in subpopulations between the two concentrations, we observed that many cells not efficiently classified as fulvestrant_low (labelled as other subpopulation) were accurately identified as sensitive to fulvestrant high. This result suggests that the accuracy of the drug response‐determined gene list leads to high efficiency in the identification of sensitive and resistant subpopulations and represents diversity in the ability of scPharm to process pharmacogenomic data with differences in quality.

### 2.5 Discovery of Dynamic Changes in the Resistant Subpopulation of PC9 Cells Treated with Erlotinib

**Figure 4:**
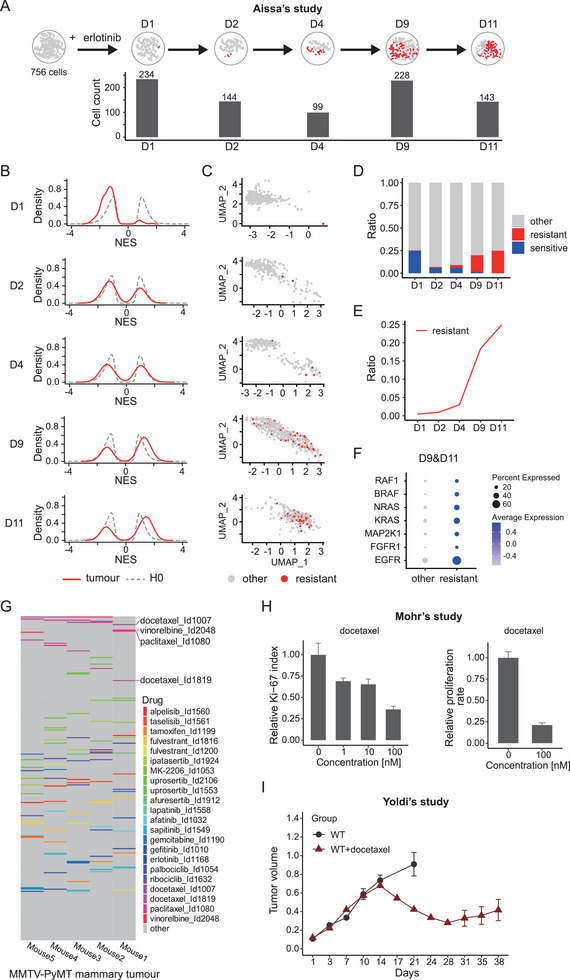
Application of scPharm in PC9 cells and MMTV‐PyMT model mice. A) Graph showing the number of drug‐resistant PC9 cells treated with erlotinib at different time points (days) identified by scPharm. Temporal cell counts were obtained from the study by Aissa et al. B) scRNA‐seq data showing the dynamics of the NES peak in PC9 cells. C) UMAP visualization highlighting the resistant cells identified by scPharm (depicted in red). D) Stacked bar chart depicting the changing proportions of sensitive, resistant and other subpopulations at different time points. E) Temporal changes in the proportions of the resistant subpopulation. F) Expression of genes in the EGFR pathway and downstream pathways. G) Heatmap showing the predicted ranking of various clinical therapeutic drugs for breast cancer treatment derived from the Opentarget Platform. The analysis is based on data obtained from five samples of the MMTV‐PyMT mouse model. H) Bar graphs showing the relative abundance of Ki‐67 (left panel) and the relative proliferation rate of breast cancer cells (right panel) in MMTV‐PyMT mice treated with different concentrations of docetaxel. The data were obtained from the study by Mohr et al. I) Diagram showing breast tumour sizes in MMTV‐PyMT mice at different time points (dark red: treated with docetaxel, grey: control). The data were obtained from the study by Yoldi et al.

The aforementioned evaluations revealed the efficacy of scPharm in identifying sensitive subpopulations in ER‐positive and HER2‐positive human breast cancer tissues. To further assess the ability of scPharm to identify resistant subpopulations and observe their dynamic changes over time, we employed scRNA‐seq data from the LUAD cell line PC9. PC9 cells harboring an exon 19 deletion in the *EGFR* gene were treated with the EGFR inhibitor erlotinib on Days 1, 2, 4, 9 and 11. Although PC9 cells were initially sensitive to EGFR inhibition, continuous treatment led to cell survival and the occurrence of drug resistance on Days 9 and 11 (**Figure** **4**A).

This dataset included a total of 1311 PC9 cells, with an average of 262 cells at each time point and 1132 genes captured per cell. The results revealed a rightward shift of the positive peak toward the *H0*_resistant distribution on Days 9 and 11, indicating the emergence of a resistant subpopulation following continuous treatment with erlotinib (Figure 4B,C). Notably, on Day 1, the negative peak shifted leftward toward the *H0*_sensitive distribution, consistent with the finding that PC9 cells initially exhibited a favourable response to erlotinib. By tracking the pharmacological subpopulations over time, we observed an increase in the resistant subpopulation accompanied by a decrease in the sensitive subpopulation (Figure 4D). Particularly noteworthy was the sharp increase in the resistant subpopulation after Day 4 (Figure 4E), which precisely coincided with the experimental findings (Figure 4A). To understand the mechanisms underlying drug resistance, we further investigated the expression of genes involved in the EGFR pathway and downstream pathways. We observed the upregulation of *EGFR*, *FGFR1*, *MAP2K1*, *KRAS*, *NRAS*, *BRAF*, and *RAF1*, indicating the reactivation of signaling pathways downstream of the inhibited EGFR in resistant PC9 cells (Figure 4F).

### 2.6 Prediction Capabilities of scPharm Across Additional Cancer Datasets

To further evaluate the applicability of scPharm, we have extended the evaluation of scPharm to include independent scRNA‐seq datasets from a broader range of cancer types, encompassing varying tumour microenvironments.

Approximately 30% of chronic myelomonocytic leukaemia (CMML) patients harbour mutations in Ras pathway genes,^[^ ^26^ ^]^ which are associated with a high risk of resistance and disease progression following treatment with hypomethylating agents (HMAs), such as azacitidine and decitabine.^[^ ^27^ ^]^ When we applied scPharm to analyse single‐cell data from these patients, azacitidine did not rank highly, further suggesting the development of resistance. Interestingly, certain ERK/MERK inhibitors (e.g., selumetinib and ERK_2440) and PI3K inhibitors (e.g., MK‐2206 and afuresertib) ranked higher (Figure S8A, Supporting Information). This observation aligns with the molecular mechanisms associated with Ras pathway mutations. In CMML patients with Ras pathway mutations, the activation of the Ras signaling pathway could increase the susceptibility of these tumours to treatments that target RAS and its downstream pathways, such as PI3K‐related inhibitors.^[^ ^28^ ^]^

In the skin cutaneous melanoma (SKCM) dataset,^[^ ^29^ ^]^ we observed that dabrafenib achieved a higher predictive ranking in the sample with the *BRAF* V600E mutation (GSM6022253) and a slightly lower predictive performance in the sample with the *BRAF* R178* mutation (GSM6022255) (Figure S8B, Supporting Information). This outcome aligns with our expectations, as dabrafenib is an FDA‐approved therapeutic agent for patients with unresectable or metastatic melanoma harboring the *BRAF* V600E mutation.^[^ ^30^ ^]^ Similarly, the drug's lowest ranking in the *HRAS*‐mutant sample (GSM6022252) is also consistent with our expectations (Figure S8B, Supporting Information). Conversely, the RAS inhibitor (THR‐103) had the highest ranking in the *HRAS*‐mutant sample and performed poorly in the *BRAF*‐mutant samples. These findings suggest that scPharm can accurately predict drug treatment effects tailored to specific samples, providing valuable guidance for therapy selection.

In another cohort of LUAD and lung squamous cell carcinoma (LUSC) datasets,^[^ ^31^ ^]^ scPharm was utilized to evaluate the ranking of samples with different mutational phenotypes in their corresponding targeted therapies, yielding favourable results (Figure S8C, Supporting Information). Among the *KRAS*‐mutant samples (GSE148466 and GSE136246), KRAS (G12C) inhibitors or MAPK inhibitors demonstrated superior rankings. This finding is consistent with the therapeutic potential of targeting RAS, a critical protein in the MAPK signaling pathway, either through direct inhibition or by targeting downstream signals.^[^ ^32^ ^]^ For the *EGFR*‐mutant samples (GSE241934), the tyrosine kinase inhibitor lapatinib, which acts as a dual inhibitor of EGFR and HER2, showed a high predicted ranking, reflecting its efficacy in treating *EGFR*‐mutant LUAD.^[^ ^33^ ^]^ Additionally, in the dataset from the PC9 cell line (GSE247684), inhibitors targeting the PI3K signaling pathway and the MAPK signaling pathway, both downstream of EGFR, were ranked favourably, indicating their potential in treating *EGFR*‐mutant LUAD.^[^ ^34^ ^]^ The echinoderm microtubule‐associated protein‐like 4–anaplastic lymphoma kinase (*EML4–ALK*) fusion gene, recognized as a critical oncogenic driver in LUAD, has led to the development of several targeted therapies, such as crizotinib and alectinib.^[^ ^35^ ^]^ In our analysis, sorafenib, another tyrosine kinase inhibitor (TKI), was ranked highly, whereas crizotinib was ranked lower. Although sorafenib primarily targets other signaling pathways (e.g., VEGFR, PDGFR, and RAF), it may exert a more pronounced inhibitory effect on *EML4–ALK*‐positive cells through nonclassical mechanisms in certain contexts.^[^ ^36^ ^]^

### 2.7 Expanding the Predictive Capabilities of scPharm to a Mouse Model

The mouse is the foremost mammalian model for studying human diseases and therapies. While scPharm primarily utilizes pharmacogenomic profiles from human cancer cell lines to predict pharmacological subpopulations, we sought to evaluate its potential applicability in a mouse model. We obtained scRNA‐seq data from a well‐established MMTV‐PyMT mouse mammary tumour model, including samples from five mice.

In total, 3504 single tumour cells, with an average of 700 cells per mouse, and 13245 genes orthologous to human genes per cell were included in our evaluation. When drugs were ranked based on their *Dr* values, chemotherapeutics such as docetaxel, vinorelbine, and paclitaxel were highly ranked among the recommended drugs, whereas targeted drugs were not (Figure 4G). This finding is consistent with the genetic background of the MMTV‐PyMT mouse model, in which ERα and PR expression decrease as the tumour progresses and HER2 is expressed at a low level. Moreover, a few in vitro studies (Figure 4H; Table S1, Supporting Information) and in vivo (Figure 4I; Table S1, Supporting Information) experiments have shown that MMTV‐PyMT mice are sensitive to docetaxel treatment, resulting in a reduction in the number of tumour cells and tumour shrinkage (Figure 4I).^[^ ^37^ ^]^ Similarly, reports on the sensitivity of the MMTV‐PyMT mouse model to paclitaxel also provide evidence supporting our results.^[^ ^38^ ^]^ These independent results provide cross‐validation of the predictive ability of scPharm in a mouse model.

### 2.8 Comprehensive Evaluation of Different Methods in Breast Cancer Samples

To comprehensively illustrate the predictive performance of scPharm, we conducted a comparative analysis with two widely used single‐cell prediction tools, scDEAL^[^ ^12^ ^]^ and CaDRReS‐Sc,^[^ ^13^ ^]^ along with the label transfer tools Scissor^[^ ^15^ ^]^ and SeuratCCA.^[^ ^18^ ^]^ Scissor and SeuratCCA are not typically used in this manner.

The evaluation of single‐cell prediction models at the individual‐cell level poses challenges since drug response assays are typically conducted on cell populations rather than on individual cells. We introduced a novel evaluation approach focused on comparing the drug recommendations generated by different tools. ER‐positive and HER2‐positive breast cancer tissue samples, which are known to be sensitive to ER or HER inhibitors, were used for this evaluation.

**Figure 5:**
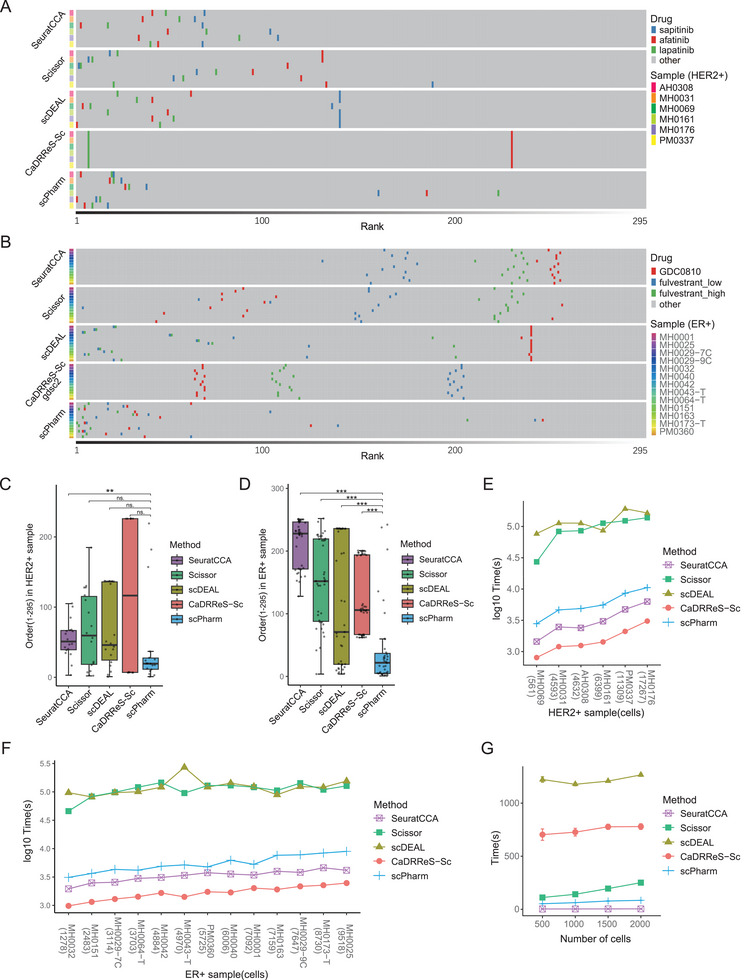
Benchmarks of different methods, including SeuratCCA, Scissor, scDEAL, CaDRReS‐Sc and scPharm. A) Predicted rankings of HER2 inhibitors in HER2‐positive samples across the different methods. B) Predicted rankings of ER inhibitors in ER‐positive samples across the different methods. C) Statistical comparison of the rankings of HER2 inhibitors in HER2‐positive samples predicted by the different methods. D) Statistical comparison of the rankings of ER inhibitors in the ER‐positive samples predicted by the different methods. The Mann‐Whitney test was employed, with “*” indicating a *p* value less than 0.05, “**” indicating a *p* value less than 0.01, and “***” indicating a *p* value less than 0.001. E) Comparison of the runtimes of different methods for HER2‐positive samples. F) Comparison of the runtimes of different methods for the ER‐positive samples. G) Comparison of different methods for calculating the runtimes of individual drugs for different single‐cell numbers sampled from a HER2‐positive breast cancer sample (PM0337).

Initially, sensitive and resistant subpopulations of breast cancer samples were predicted using different tools. We then employed the *Dr* values developed by scPharm to rank all drugs in the analysis (see the Experimental Section) (**Figure** **5**A,B). Our focus was on examining the rankings of three HER2 inhibitors, afatinib, lapatinib, and sapitinib, in HER2‐positive breast cancer samples. For methods such as CaDRReS‐Sc, which directly outputs drug rankings, we used the provided rankings. For methods such as scDEAL, Scissor, and SeuratCCA, which output sensitive/resistant classifications, we calculated the *Dr* value. The findings indicated that, except for sample MH0161, scPharm consistently ranked these drugs at the top and near each other (Figure 5A), significantly outperforming the other tools (Figure 5C). While CaDRReS‐Sc exhibited consistent prediction results across different samples, substantial differences were observed between the samples.

Due to the default use of GDSC1 drugs for calculations by CaDRReS‐Sc, no prediction was available for the drug sapitinib. To address this issue, we extended CaDRReS‐Sc to utilize GDSC2 data for training and prediction, enabling a comparison of predictions for ER‐positive breast cancer samples. In these samples, the rankings of the ER inhibitors GDC0810 and fulvestrant were similarly prominent and concentrated in scPharm (Figure 5B), significantly surpassing those of the other tools (Figure 5D).

We subsequently assessed the computational time of the tools. Notably, the efficiency of scPharm was markedly greater than that of Scissor and scDEAL, albeit slightly lower than that of CaDRReS‐Sc and SeuratCCA (Figure 5E,F). CaDRReS‐Sc achieved the highest efficiency, predicting responses for all drugs in a single run, whereas other tools relied on iterative processes. When focusing on only one sample, such as the HER2‐positive breast cancer sample PM0337, scPharm completed a pharmacological subpopulation analysis for a single drug within 1 min across a range of 500 to 2000 cells, which was significantly less time‐consuming than that of all other tools, except SeuratCCA (Figure 5G). Furthermore, we observed runtime stability across multiple repetitions for the sample, whereas CaDRReS‐Sc and scDEAL exhibited slight fluctuations in runtime (Figure 5G). Additionally, the runtime of all tools increased with an increasing number of cells.

### 2.9 Identification of Potential Combination Regimens

**Figure 6:**
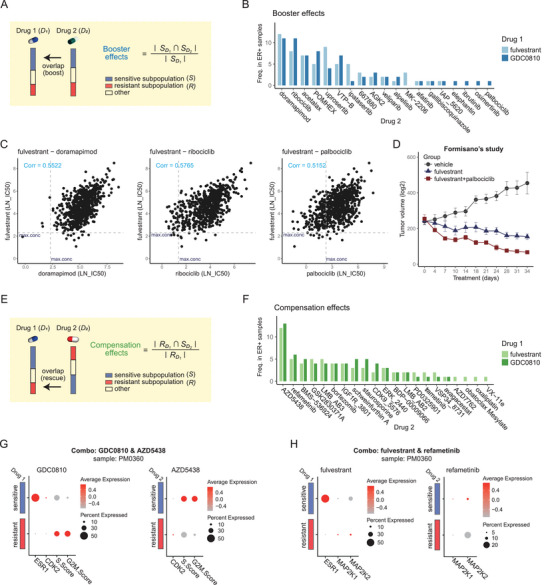
Application of scPharm in drug combinations for the treatment of ER‐positive breast cancer. A) Schematic and formula used to calculate the booster effect. B) Frequencies of the top 5 medication pairs in each sample predicted by the booster effect across the 13 samples. C) Scatterplot of the Pearson correlation coefficients of the IC50 values for the three drug combinations in all cancer cell lines. The dashed line indicates the maximum concentration of the drug. D) Diagram of the tumour size after treatment with fulvestrant in combination with palbociclib at different time points. Data were obtained from the study by Formisano et al. E) Schematic and formula used to calculate the compensation effects. F) Frequencies of the top 5 medication pairs in each sample predicted by the compensation effects across the 13 samples. G) Plots of gene expression and functions indicating the drug combinations (GDC0810 and AZD5438) in sample PM0360. H) Plot of gene expression indicating the drug combination (fulvestrant and refametinib) in sample PM0360.

scPharm can be utilized to formulate potential drug combinations using the set covering method, which encompasses booster and compensation effects (Figure 1). Booster effects involve the use of two distinct drugs with different targets to eliminate a specific subpopulation of tumour cells (**Figure** **6**A). After applying scPharm to the ER‐positive breast cancer data, we extracted the top 5 booster effects of ER inhibitors (fulvestrant and GDC0810) on each sample, along with their combinations with other drugs. By investigating these booster effect‐based drug combinations, we found that drugs such as doramapimod and ribociclib were commonly identified in more than 10 samples (out of a total of 13 samples) (Figure 6B). The correlation of the IC50 values of drug pairs in all cancer cell lines was calculated to better understand the booster effects between drug pairs. The results revealed that both the most frequently occurring drugs, doramapimod and ribociclib, as well as the less frequent drug palbociclib, were strongly correlated with ER‐targeted drugs, with values exceeding 0.5 (Figure 6C; Figure S9, Supporting Information). Previous studies have documented the significant tumour‐regressive effects of fulvestrant and palbociclib/ribociclib on ER‐positive breast cancer (Figure 6D).^[^ ^39^ ^]^ Ribociclib and palbociclib are both CDK4/6 inhibitors with similar mechanisms of action and effects, making them clinically applicable for the treatment of ER‐positive breast cancer.^[^ ^39^, ^40^ ^]^ This result strongly supports the credibility of our predicted drug combinations with fulvestrant or GDC0810. The top‐ranked drug, doramapimod, targets JNK and p38 signaling and is currently only used in clinical trials for psoriasis and rheumatoid arthritis. However, we speculate that this drug has potential for use in adjuvant therapy in patients with ER‐positive breast cancer and could be another possible combination for treating acute myeloid leukaemia.^[^ ^41^ ^]^

Compensation effects indicate that drug 2 can serve as a complementary therapeutic approach to drug 1 (Figure 6E). Based on the magnitude of the compensation effects, the top 5 drug combinations were extracted for each sample of patients with ER‐positive breast cancer. AZD5438, which targets CDK2,^[^ ^42^ ^]^ appeared in the predicted drug combinations for all the samples (Figure 6F). The analysis revealed significantly greater cell cycle activity in cells resistant to drug 1 (GDC0810) and in cells sensitive to drug 2 (AZD5438) (Figure 6G). This finding indicates that drug 2 rescues the resistance of cells to drug 1 by inhibiting the ectopic activation of the cell cycle in the subpopulation resistant to drug 1. Additionally, refametinib, an MEK1/2 inhibitor,^[^ ^43^ ^]^ was predicted to have a combined effect on more than 6 samples. Similarly, in fulvestrant‐resistant cells, *MAP2K1* and *MAP2K2* were significantly upregulated (Figure 6H). The above results indicate the existence of compensation effects and the efficacy of combination pairs predicted by scPharm.

### 2.10 Prediction of Drug Side Effects on Healthy Human Tissues

scPharm is designed to evaluate drug side effects by utilizing healthy cells from the tumour microenvironment. In the scPharm analysis pipeline, healthy cells were distinguished from tumour cells via the CopyKAT method.

**Figure 7:**
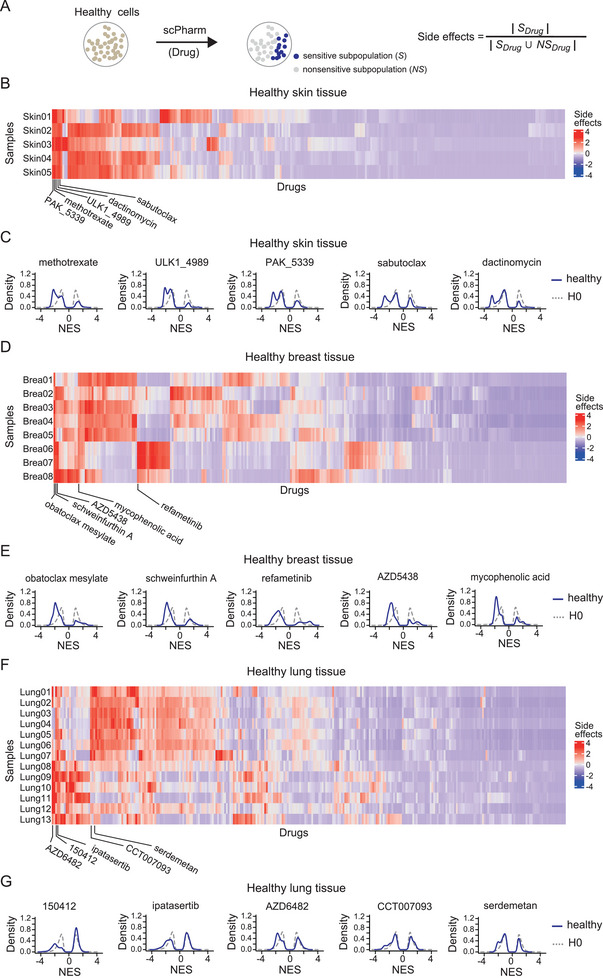
Prediction of side effects of drugs on healthy human tissues. A) Index of the side effects evaluation. B) Heatmap showing the side effects of all drugs on healthy skin tissues from different donors. C) Density plot depicting the distributions of NESs for the 5 drugs with the highest indices of side effects in pooled healthy skin tissues. D) Heatmap showing the side effects of all drugs on healthy breast tissues from different donors. E) Density plot depicting the distributions of the NESs for the 5 drugs with the highest indices of side effects in pooled healthy breast tissues. F) Heatmap showing the side effects of all drugs on healthy lung tissues from different donors. G) Density plot depicting the distributions of the NESs for the 5 drugs with the highest indices of side effects in pooled healthy lung tissues.

To assess potential side effects, we used an index representing the ratio of healthy cells in the sensitive subpopulation to all healthy cells (**Figure** **7**A). Using scPharm, we identified sensitive subpopulations from the scRNA‐seq data of healthy human tissues, including 5 skin, 8 breast and 12 lung tissue samples, generating a comprehensive landscape of potential side effects for all drugs (Figures 7B–G). Notably, we observed substantial heterogeneity of side effects both within samples of the same tissue and across different tissues (Figure 7B,D,F).

For skin tissues, the 5 drugs with the most severe potential side effects were methotrexate, ULK1_4989, PAK_5339, sabutoclax, and dactinomycin (Figure 7B). The NES distribution curves of single healthy cells exhibited dual negative peaks, with one negative peak significantly shifting toward the *H0*_sensitive distribution, indicating the presence of a subpopulation significantly sensitive to these drugs (Figure 7C). Methotrexate is an antimetabolite and antifolate drug. This drug works by inhibiting dihydrofolate reductase, an enzyme involved in the synthesis of DNA, RNA, and proteins. Methotrexate is known to cause various skin‐related side effects, including alopecia, radiation recall, sunburn reactivation, and photosensitivity. Dactinomycin, also known as actinomycin D, is a chemotherapy medication used to treat numerous types of cancer. Dactinomycin is known to cause various skin‐related side effects, including rash, pigment changes, photosensitivity, and even more severe skin reactions, such as Stevens–Johnson syndrome and toxic epidermal necrolysis.^[^ ^44^ ^]^

For breast tissues, the 5 drugs with the greatest side effects were obatoclax mesylate, schweinfurthin A, refametinib, AZD5438, and mycophenolic acid (Figure 7D). The leftward shift of the negative peak toward the *H0*_sensitive distribution for these drugs (Figure 7E) suggested potential toxicity to mammary gland cells. Compared with healthy skin and breast tissues, healthy lung tissues presented milder side effects, with even the top 5 drugs showing negative peaks with a slight leftward shift (Figure 7F,G), indicating that most single cells do not respond significantly to these drugs.

## 3 Discussion

The challenge of molecular heterogeneity within tumours poses a significant obstacle to the effectiveness of anticancer treatments. Tumours characterized by high intratumoural heterogeneity often lead to unfavourable clinical outcomes, including drug resistance and a poor prognosis. Thus, the precise identification of tumour heterogeneity is crucial for the development of successful therapeutic interventions. scRNA‐seq technology provides a means to capture gene expression profiles at single‐cell resolution, whereas drug perturbation experiments yield valuable drug response data at the bulk cell level. Through the integration of these multimodal datasets, computational methods can be used to effectively transfer drug response information from bulk cells to individual cells. This integration has the potential to provide a solution for elucidating the complexities of intratumour therapeutic heterogeneity.

In this study, we introduced scPharm, a computational framework designed to integrate large‐scale pharmacogenomic profiles with scRNA‐seq data. The primary objective was to discern intratumour therapeutic heterogeneity at single‐cell resolution. scPharm was used to calculate the Pearson correlations between the pretreatment gene expression levels and the AUC values obtained after 72 h of treatment and generated a drug response‐determined gene ranking list. This list reflects pretreatment gene expression levels and is independent of specific time points after drug exposure. Therefore, scPharm specifically focused on identifying baseline gene expression patterns that correlate with drug sensitivity and resistance. These baseline patterns are valuable for understanding inherent differences in drug responses across cell lines, regardless of time‐dependent fluctuations after treatment. In this context, scPharm is designed to identify inherent pretreatment sensitivity and resistance signatures, but it is not currently equipped to predict the exact time point at which resistant or sensitive cell populations begin to diverge during treatment.

The framework's statistical methodology addresses the challenges of current deep learning models applied in this domain. These challenges include coping with small sample sizes, optimizing label binarization, leveraging data across modalities, and enhancing biological interpretation. Additionally, scPharm extends beyond the prioritization of tailored drugs for a tumour. scPharm is notable for its unique ability to predict combination strategies, gauging compensation effects or booster effects between two drugs. scPharm can be extended to identify combinations of more than two drugs. For example, if the resistant subpopulation of the first drug is complemented by both the sensitive subpopulations of the second and third drugs, the third drug can be included in the combination. In essence, scPharm generates a sorted list of potential drugs for combination, and in our current analysis, we presented only the top‐ranked drugs for each combination.

Moreover, scPharm has been applied to single‐cell sequencing data obtained from real‐world tumour tissues. These scenarios involve a mixture of various cell types within the tumour microenvironment rather than focusing solely on pure cancer cells. By directing attention to these “normal” cells, scPharm has the capacity to evaluate potential drug toxicity or side effects on nontumour cells. Gene signatures derived from cancer cell lines may not perfectly reflect the behaviour of healthy tissues. However, due to the lack of available pharmacological data on healthy cells, we applied the drug response‐determined gene list generated from cancer cell lines to predict drug toxicity in healthy tissues. While this approach may not be as direct as using healthy cells themselves, we hypothesize that certain sensitivity and resistance gene signatures could be shared between cancerous and healthy cells. This capability provides valuable insights into the comprehensive effects of a given drug on the entire tumour tissue.

We conducted a thorough comparative analysis to assess the predictive performance of scPharm against other single‐cell prediction tools, including scDEAL and CaDRReS‐Sc, as well as label transfer methods such as Scissor and SeuratCCA. While scDEAL employs a deep transfer learning approach to integrate bulk cell line data with scRNA‐seq data, CaDRReS‐Sc is a machine learning framework designed for predicting drug responses. Notably, Scissor and SeuratCCA are not typically used in this manner; Scissor is a method for identifying cell subpopulations associated with a given phenotype, and SeuratCCA is a popular label transfer method within the Seurat package. Our systematic evaluation revealed the superior predictive performance and computational efficiency of scPharm. scPharm differs from these tools in several key aspects. 1) Unlike scDEAL and CaDRReS‐Sc, which are machine learning‐based, scPharm is a statistical methodology that calculates drug response using the static NES. This statistical approach offers an advantage in terms of computing time, as it does not require large‐scale training across multiple samples. 2) scPharm is specifically designed to account for the complexity of real‐world tumour tissues, which often consist of a mixture of various cell types within the tumour microenvironment. This property enables scPharm to provide more biologically relevant insights. 3) scPharm is more precise in its predictions, as it generates drug response‐determined gene ranking lists using only specific cell lines that correspond to the tissue source of the single‐cell data. In contrast, machine learning‐based methods, such as scDEAL, often need to combine data from all cancer cell lines due to the requirement for large sample sizes for training. 4) CaDRReS‐Sc directly outputs drug rankings, and other methods, such as scDEAL, focus on binary sensitive/resistant classifications, which may overlook the broader clinical context, such as drug prioritization and potential side effects. scPharm, on the other hand, provides valuable insights into tailored drug prioritization and combination strategies and even allows for the potential assessment of drug toxicity.

Additionally, we evaluated the potential for false‐positive associations of scPharm by conducting experiments using randomized bulk data, where both the cell lines and gene expression profiles were shuffled to simulate a scenario with no real biological associations. The results showed that few of sensitive/resistant cells were identified when randomized bulk data was used. This demonstrates that scPharm robustly handles randomized inputs and minimizes the risk of false‐positive associations, reinforcing the reliability of the predictions in real biological contexts (Figure S10A, Supporting Information).

Given the advantages of scPharm, several points need to be addressed. First, one key strength of scPharm is rooted in the robust positive correlation between NESs and AUC values, as depicted in Figure 2. To reduce the effect of tissue‐specific expression on the predictions, drug response‐determined gene lists were generated using cell lines from the same cancer type. By investigating 12 types of cancer, we observed robust correlations that were consistently present across pancancer samples and a diverse array of drugs. The core principle behind using NESs to predict drug response relies on calculating the NES from scRNA‐seq data and retrieving the AUC from the cell population of the same cancer cell lines. While a positive correlation was still observed when the NES was calculated based on bulk RNA‐seq data, it was not as strong as that calculated from single‐cell data (Figure S10B, Supporting Information). This difference may be attributed to the efficiency of the MCA method in extracting Cell‐ID from millions of single cells compared with bulk cell samples. Therefore, scPharm exploits the data structure of scRNA‐seq to improve the correlation between NESs and drug responses.

Second, the evaluation of single‐cell prediction models at the individual‐cell level poses challenges since drug response assays are typically conducted on cell populations rather than on individual cells. Currently, some publicly available works still use bulk cell data to evaluate the performance of models. We introduced a novel evaluation approach to address this issue. Instead of relying on evaluations using bulk cell data, we employed two single‐cell RNA‐seq datasets, namely, ER‐positive and HER2‐positive breast cancer tissue samples. These samples, which are known to be sensitive to ER or HER inhibitors, allowed us to rank all drugs based on their corresponding drug sensitivity. The high rankings of ER or HER inhibitors indicate good predictive performance. This innovative approach is a step forwards in evaluating the predictive performance on single‐cell samples, especially when drug response labels are not readily available.

Finally, while the current version of scPharm excels in predicting potential drug toxicity or side effects on “normal” cells in tumour tissue, its potential can be extended to large‐scale investigations based on large sample sizes, as well as the precise classification of “normal” cells in the tumour microenvironment, such as T cells, B cells and fibroblasts. This expansion will enable a more comprehensive understanding of the impacts of drugs on both the tumour itself and its microenvironment.

In summary, scPharm is a computational framework tailored for scRNA‐seq data that integrates pharmacogenomic profiles to identify therapeutic heterogeneity within tumours at single‐cell resolution. This tool not only prioritizes tailored drugs but also provides insights into combination therapy regimens and drug toxicity in cancers.

## 4 Experimental Section

### Data Source

The GDSC dataset includes genomic data from hundreds of cancer cell lines, along with their responses to a wide range of anticancer drugs. The data capture the sensitivity of these cell lines to various compounds and link this information to the genetic makeup of the cells, including mutations, copy number variations, and gene expression profiles prior to treatment. Our study utilized bulk RNA‐seq data (gene expression profiles) from 969 cancer cell lines covering more than 30 cancer types prior to treatment, along with their drug responses to 295 drugs, including 242036 drug response measurements.

The bulk RNA‐seq data were obtained from the Cell Model Passports database (https://cellmodelpassports.sanger.ac.uk/). Drug response data were obtained from the GDSC2 project in the GDSC database (https://www.cancerrxgene.org/, release 8.4, July 2022).^[^ ^5^ ^]^ The drug response was quantified by calculating the AUC and the half maximal inhibitory concentration (IC50) after 72 h of treatment, with higher values indicating drug resistance and lower values indicating drug sensitivity in cancer cell lines. The scRNA‐seq data of human cancer cell lines were collected from the SCP542 dataset via the Single Cell Portal (https://singlecell.broadinstitute.org/single_cell). Single‐cell data from 12 cancers were used in this study.

scRNA‐seq data were obtained from the National Center for Biotechnology Information (NCBI) Gene Expression Omnibus (GEO) database, specifically from the GSE161529, GSE158677 and GSE134839 datasets, to evaluate our model. These datasets included scRNA‐seq profiles for 6 HER‐positive and 13 ER‐positive breast tumours, 5 mice from the MMTV‐PyMT mouse mammary tumour model, and the LUAD cell line PC9 treated with erlotinib on Days 1, 2, 4, 9 and 11, respectively. Additional independent scRNA‐seq datasets with a broader range of cancer types (SKCM, CMML, LUSC, etc.) were used for the extended evaluation. The details of all these scRNA‐seq data are in Table S2 (Supporting Information).

For statistical analyses, scRNA‐seq data from GSE196638, GSE122960, GSE150247, GSE164898 and GSE151177 were used to construct null distributions (*H0*). These datasets include scRNA‐seq profiles from healthy human lung, skin, and breast tissues (Table S3, Supporting Information). The datasets were also utilized to evaluate the potential side effects of drugs. Pathway activity was calculated by collecting canonical pathways from the Kyoto Encyclopedia of Genes and Genomes database. All source data are provided with this article.^[^ ^45^ ^]^

### Data Processing

For the bulk RNA‐seq data, gene expression profiles are presented using the normalized expression index—transcripts per million (TPM)—with the removal of missing values and genes that showed no expression across all cell lines. Notably, only cell lines with corresponding drug response information were retained for analysis. For the scRNA‐seq data, the R package Seurat (v4.3.0)^[^ ^46^ ^]^ was used for data processing, including reads and initial quality control. All Seurat preprocessing steps were performed using the default parameters. Specifically, quality control was conducted by filtering genes that were not present across a minimum of 3 cells (min.cells = 3) and by filtering cells that did not contain a minimum of 200 genes (min.features = 200), and maxGene and maxUMI were used to filter the outlier cells in the datasets GSE161529, GSE134839, and GSE136246 (Tables S2 and S3, Supporting Information), to ensure more cells were included in the analysis. The processed data were further followed by normalization and dimensionality reduction clustering.

### scPharm Framework

scPharm is a comprehensive framework composed of two interconnected modules designed for the analysis and prediction of pharmacological subtypes of single cells, as well as the prioritization of tailored drugs and exploration of combinatorial drug usage and potential side effects (Figure 1). Moreover, scPharm specifies a specific cancer type that matches the scRNA‐seq data and the bulk RNA‐seq data. This matching ensures that scPharm interprets the therapeutic heterogeneity within specific tumour types, optimized for both performance and biological relevance.

Module 1 of scPharm utilizes gene expression profiles from cancer cell lines determined by bulk RNA‐seq and their corresponding drug response data from the Cell Model Passports and GDSC databases. The Pearson correlation method was used to calculate the correlations between gene expression and drug responses, and a list of genes ranked according to drug response was generated for each drug within a specific cancer type. Genes at the top of the ranked list whose expression was positively correlated with the AUC were associated with drug resistance, whereas those at the bottom were associated with drug sensitivity.

In real‐world scenarios where scRNA‐seq data are derived from tumour tissues of the same cancer type as the bulk RNA‐seq data, rather than from cancer cell lines, scPharm employs CopyKAT to differentiate between cancer cells and healthy cells based on gene copy number variations.^[^ ^19^ ^]^ Cancer cells are used to identify pharmacological subpopulations and rational medicines, whereas healthy cells are employed to assess potential drug side effects. MCA is utilized to extract gene signatures from individual cells, effectively defined as Cell‐ID.^[^ ^20^ ^]^ Leveraging the drug response‐determined gene list and Cell‐ID as inputs, GSEA^[^ ^17^ ^]^ evaluates whether a single cell is sensitive to a drug according to the distribution of its Cell‐ID throughout the ranked gene list for the specific drug. If the Cell‐ID of an individual cell is predominantly enriched at the top of the list, the cell is predicted to be resistant to the tested drug, whereas enrichment at the bottom suggests sensitivity. The NES obtained through GSEA indicates the drug response of a single cell, categorizing it as sensitive, resistant, or other.

To model the null distributions of NESs in the context of drug responses, scRNA‐seq data from healthy human lung, skin and breast tissues were collected. The null distribution was constructed using healthy human tissues because this approach better represents the differences between tumour and normal cells, which is crucial for identifying tumour‐specific vulnerabilities. Each tissue sample was integrated into a single‐cell sample using the RunHarmony method in harmony package.^[^ ^47^ ^]^ Quality control, normalization, dimensionality reduction, clustering, MCA and GSEA on each tissue sample is subsequently conducted. After completion, the NESs for all cells related to each drug were merged to serve as the null distribution for this study. Gaussian mixture model decomposition was performed using the normalmixEM method in the Mixtools package to accurately estimate the parameters of the null distribution.^[^ ^21^ ^]^ Using a unified threshold based on the mean ± one standard deviation, scPharm categorized tumour single‐cell data into sensitive and resistant subpopulations for a given drug, increasing the interpretability and reliability of the framework.

Module 2 of scPharm evaluates the suitability of drugs within a specific tumour sample by defining a score, denoted Dr, based on the number of sensitive and resistant single cells obtained from Module 1. The score is calculated as *Dr*  = *R_s_* *(1  −  *R_r_*), where *R_s_* represents the ratio of the sensitive subpopulation and *R_r_* represents the ratio of the resistant subpopulation in the tumour sample. Based on this score, scPharm ranks drugs to provide drug recommendations.

scPharm was also used to explore potential combinatorial drug therapies with compensation and booster effects. Compensation involves sensitization to a second drug in drug‐resistant cells that are unresponsive to the first drug, thereby compensating for the lack of response. In contrast, booster effects occur when the second drug synergistically enhances the cytotoxic effect of the first drug on sensitive tumour cells. The set covering method was used to quantify these combination effects. The compensation effects were calculated with the following Equation (1):

(1) $$Compensation\;effects=\frac{\left|\;R_{D1}\;\cap \;S_{D2}\;\right|}{\left|\;R_{D1}\;\right|}$$

Booster effects were calculated with the following Equation (2):

(2) $$Booster\;effects=\frac{\left|\;S_{D1}\;\cap S_{D2\;}\right|}{\left|\;S_{D1}\;\right|}$$

where *R*_*D*1_ represents the cell subpopulation resistant to Drug 1, *S*_*D*2_ represents the cell subpopulation sensitive to Drug 2, and *S*_*D*1_ represents the cell subpopulation sensitive to Drug 1.

Additionally, scPharm considered healthy cells infiltrating the tumour microenvironment in the analysis. If these healthy cells were also sensitive to a drug, the drug has potential toxicity to healthy cells and is classified as having side effects. To quantify the side effects of a drug on a specific patient, the index was constructed and described by the following Equation (3):

(3) $$Side\;\;effects\;=\;\frac{\left|\;S_{Drug}\;\right|}{\left|\;S_{Drug\;}\cup \;NS_{Drug}\;\right|}$$

where *S_Drug_* represents the cell subpopulation sensitive to *Drug* among healthy cells and where *NS_Drug_* represents the cell subpopulation insensitive to *Drug*.

### Evaluation of scPharm

Two single‐cell drug sensitivity prediction tools were used to evaluate scPharm: scDEAL^[^ ^12^ ^]^ and CaDRReS‐Sc.^[^ ^13^ ^]^ The label transfer methods Scissor^[^ ^15^ ^]^ and SeuratCCA^[^ ^18^ ^]^ were also utilized to transfer the response labels of the pharmacogenomic data to the single‐cell tumour data.

scDEAL is a deep learning‐based method that uses neural networks to predict drug sensitivity in single cells. Using deep transfer learning, scDEAL trains and optimizes a model with drug response information from bulk data, embeds both single‐cell and bulk data into a shared low‐dimensional feature space, and transfers knowledge and relation patterns from bulk data to single‐cell data to identify how single cells might respond to specific drug treatments. In our evaluation, the scDEAL code was executed with the default parameters for the scRNA‐seq data and drugs, and the predicted drug response in scDEAL, sens_label (1: sensitive, 0: resistant), was extracted for evaluation.

CaDRReS‐Sc is a machine learning‐based drug recommendation system. It constructs a “drug‒genome space” to capture relationships between drugs and various sample types, including single‐cell clusters, enabling the prediction of drug sensitivity at the single‐cell level. Additionally, it ranks drugs by predicting their efficacy in cancer cells using models trained on drug sensitivity data, making it a useful tool for drug prioritization. In our evaluation, drug sensitivity in single cells was predicted using the default parameters, and the calculated cell death rate (cell_death) for each drug was extracted as the ranking result for evaluation. Considering that CaDRReS‐Sc defaults to use GDSC1 data, it was additionally expanded the training and prediction of CaDRReS‐Sc to include GDSC2 data to facilitate a comparison of the results (named CaDRReS‐Sc.gdsc2).

Scissor is a method that leverages single‐cell data to identify cell subpopulations associated with a given phenotype. It quantifies the similarity between individual cells and bulk samples via a Pearson correlation matrix by integrating phenotype‐associated bulk expression data with single‐cell data. Scissor then optimizes a regression model using the correlation matrix and phenotypic data to pinpoint cell subpopulations associated with the phenotype. In our evaluation, Scissor was employed to transfer labels from the pharmacogenomic data to the scRNA‐seq data. For each drug‐treated bulk cancer cell line, the cell lines were first divided into sensitive and resistant cells based on the maximum drug concentration using IC50 values, which served as the phenotypic input for Scissor. Scissor was subsequently utilized in binomial mode to predict tumour cells.

SeuratCCA, a label transfer method in the Seurat package, allows the integration of single‐cell measurements across different scRNA‐seq technologies and modalities. Bulk data from cancer cell lines were binarized into sensitive and resistant labels based on the maximum drug concentration using IC50 values. The FindTransferAnchors method was then used to compute anchors between the cancer cell line expression profiles and the scRNA‐seq data. Finally, the MapQuery method was used to transfer the bulk cancer cell line labels to the scRNA‐seq data.

Given the lack of gold standard data for predicting single‐cell drug responses using traditional benchmark methods, A novel evaluation approach was introduced and focused on comparing the drug recommendations generated by different tools. CaDRReS‐Sc calculated drug recommendation rankings based on cancer cell kill scores, but the remaining tools calculated drug recommendation rankings using the *Dr* values defined in scPharm. Systematic comparisons of scRNA‐seq data from HER2‐positive and ER‐positive breast cancer samples were performed. These samples are known to be sensitive to HER inhibitors or ER inhibitors. All evaluations were conducted using the same system configuration, with a CPU core limit of 20.

### Statistical Analysis

A one‐sided Mann‒Whitney test was conducted to compare NESs between the sensitive group and resistant group classified by AUC values.^[^ ^48^ ^]^ If the NESs in the resistant group were greater than those in the sensitive group and the *p* value was less than 0.1, it was concluded that the difference was significant.

The significance of differences in pathway activity between HER2‐positive breast cancer samples was assessed using the Mann‒Whitney test. The symbols “ns”, “*”, “**”, “***” and “****” represent significance levels of 0.05 ≤ *p* < 1, 0.01 ≤ *p* < 0.05, 0.001 ≤ *p* < 0.01, *p* < 0.001, and *p* < 0.0001, respectively.

Single‐cell subpopulations of the same drug were compared at different maximum screening concentrations by conducting a one‐sided Mann‒Whitney test. The symbols “ns”, “*”, “**” and “***” represent significance levels of 0.05 ≤ *p* < 1, 0.01 ≤ *p* < 0.05, 0.001 ≤ *p* < 0.01, and *p* < 0.001, respectively.

## Conflict of Interest

The authors declare no conflict of interest.

## Author Contributions

P.T. and J.Z. are Co‐first authors. H.W. conceived the hypothesis. P.T., J.Z., and H.W. designed and performed the data analysis. K.Q., Y.F., Y.X., T.W., S.C., Y.Z., and B.Z. collected and preprocessed the data. C. A. participated in helpful discussions. P.T., J.Z., and H.W. interpreted the results and wrote the manuscript.

## Supporting information



Supporting Information

## Data Availability

The data that support the findings of this study are available in the supplementary material of this article.
