# Supplementary material for: scPharm: Identifying Pharmacological Subpopulations of Single Cells for Precision Medicine in Cancers
Source: Adv Sci (Weinh). 2024 Nov 19;12(2):2412419. doi: 10.1002/advs.202412419 (PMC11727242; doi:10.1002/advs.202412419)
Supplement: Supplementary file 1 — Supporting Information [file ADVS-12-2412419-s001.docx]

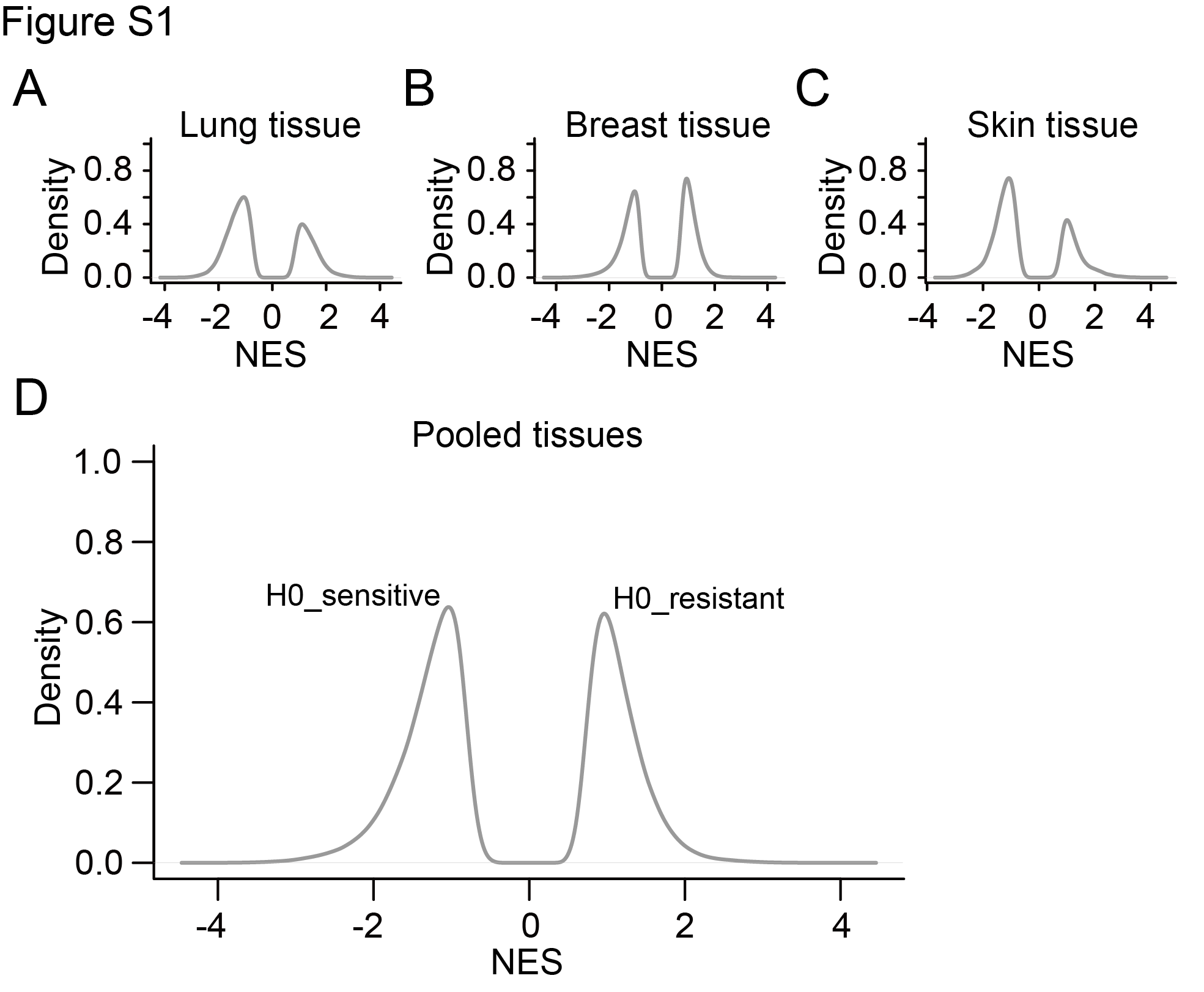


**Figure S1. Null distribution of the NES.** **A.** Density plot depicting the NESs of single cells from healthy lung tissues across all drugs. **B.** Density plot depicting the NESs of single cells from healthy breast tissues across all drugs. **C.** Density plot depicting the NESs of single cells from healthy skin tissues across all drugs. **D.** Density plot depicting the NESs of single cells from pooled healthy tissues across all drugs.


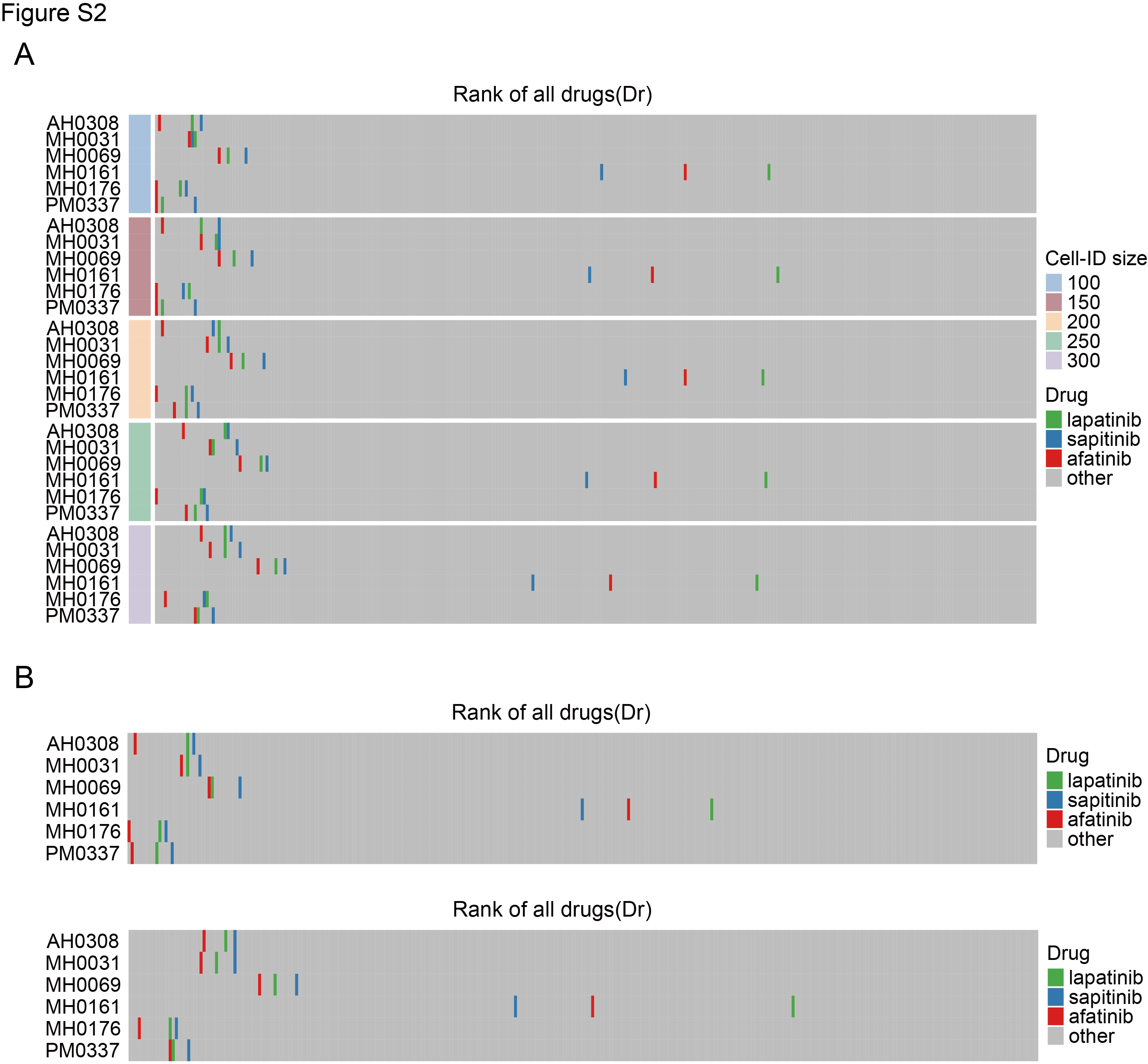


**Figure S2.** **The optimization of the parameters of scPharm.** **A.** Quantitative gradient experiment of the Cell-ID size. Rankings of HER2 inhibitors in HER2-positive breast cancer samples across different cell ID sizes (100, 150, 200, 250, and 300) are shown. **B**. Comparison of NES thresholds. Top panel: Ranking of HER2 inhibitors in HER2-positive breast cancer samples when the NES threshold was based on the null distribution. The Cell-ID size was set to 200. Bottom panel: Ranking of HER2 inhibitors in HER2-positive breast cancer samples when the NES threshold was based on the gene-wise perturbation. The Cell-ID size was set to 200.


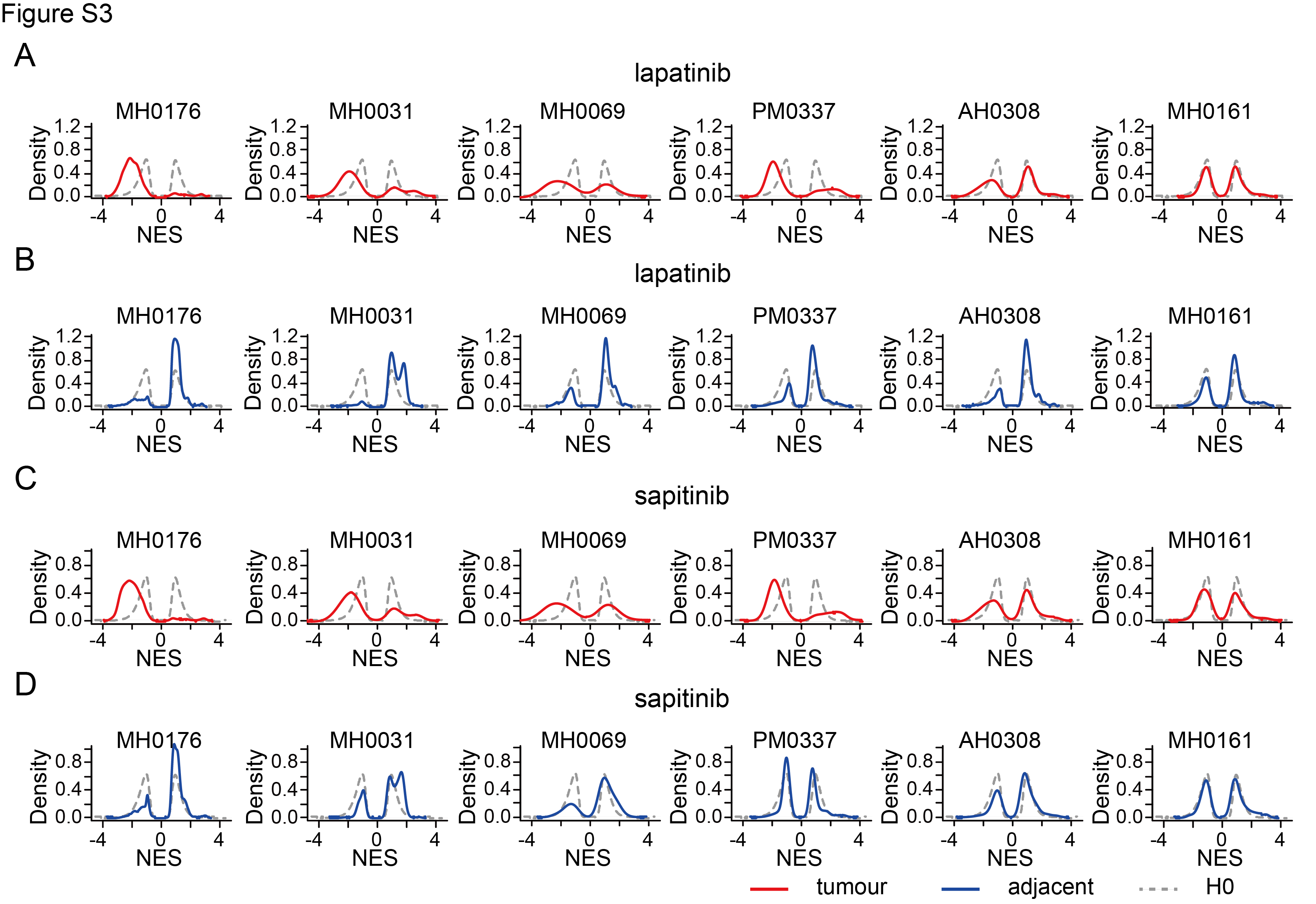


**Figure S3. Application of scPharm to HER2-positive breast cancer.** **A.** Density plot depicting the NESs of single cells from 6 HER2-positive breast cancer tissues (red curves) and healthy human tissues (grey curves) for lapatinib. **B.** Density plot depicting the NESs of single cells from tumour-adjacent tissues and healthy human tissues for lapatinib. **C.** Density plot depicting the NESs of single cells from 6 HER2-positive breast cancer tissues (red curves) and healthy human tissues (grey curves) for sapitinib. **D.** Density plot depicting the NESs of single cells from tumour-adjacent tissues and healthy human tissues for sapitinib.


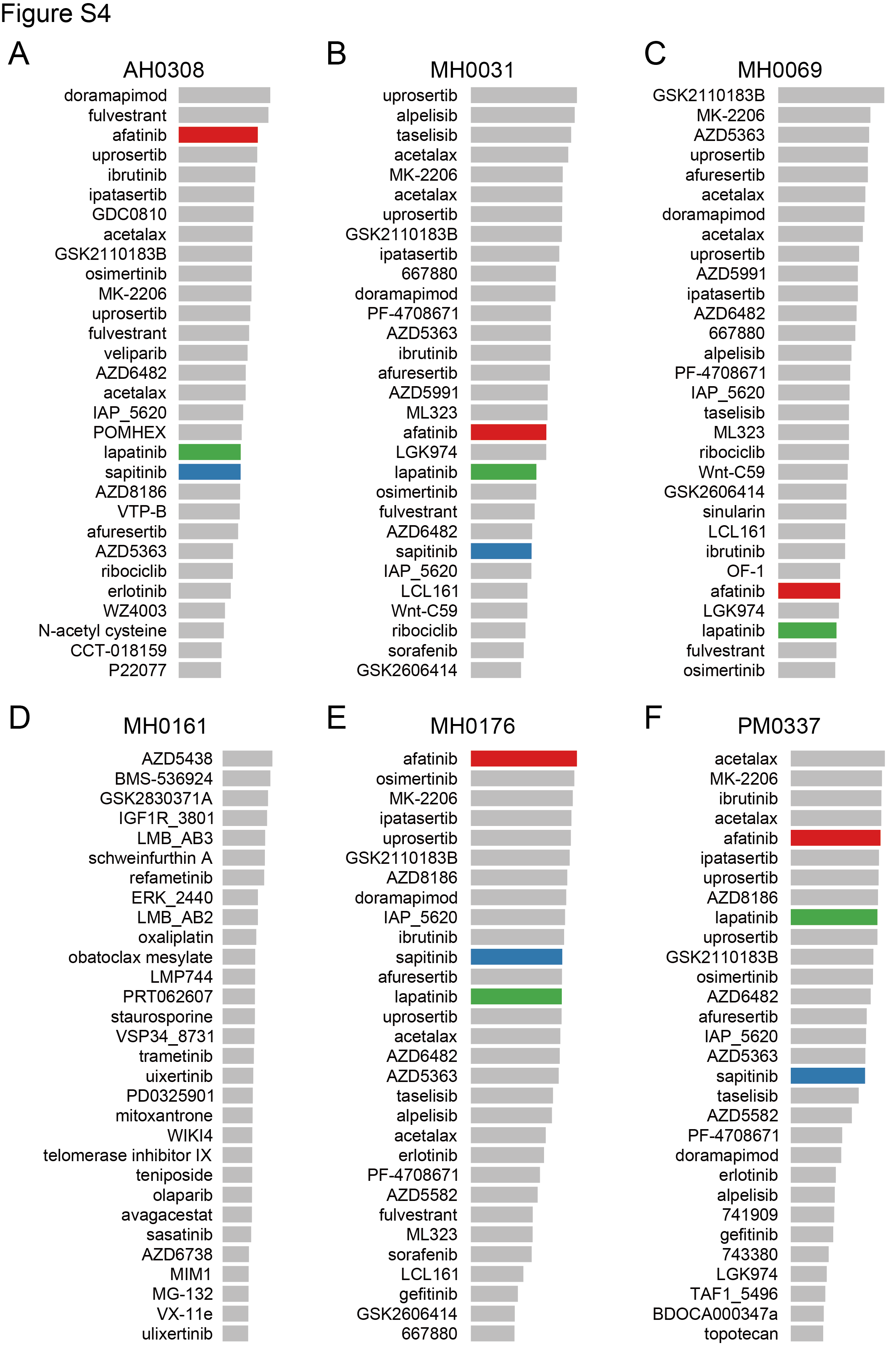


**Figure S4. The recommended drugs for HER2-positive samples.** **A-F.** The top 30 recommended drugs for AH0308, MH0031, MH0069, MH0161, MH0176 and PM0337. The length of the bar represents the level of effectiveness.

**
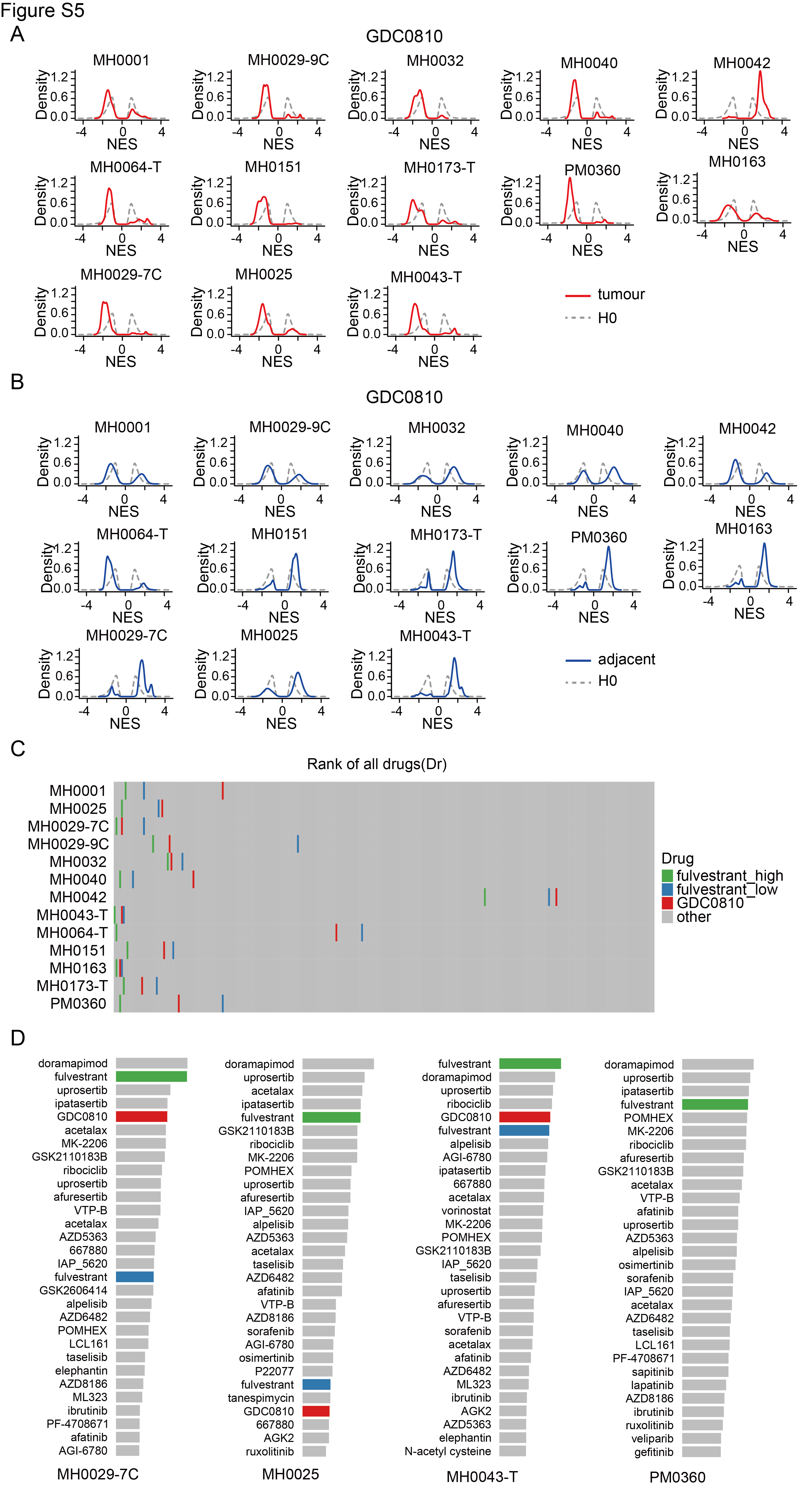
**

**Figure S5. Application of scPharm to ER-positive breast cancer.** **A.** Density plot depicting the NESs of single cells from 13 ER-positive breast cancer tissues (red curves) and healthy human tissues (grey curves) in the context of GDC0810. **B.** Density plot depicting the NESs of single cells from tumour-adjacent tissues and healthy human tissues in the context of GDC0810. **C.** Ranking of the two ER inhibitors in the ER-positive samples. **D.** The top 30 recommended drugs for MH0029-7C, MH0025, MH0043-T and PM0360. The length of the bar represents the level of effectiveness.

**
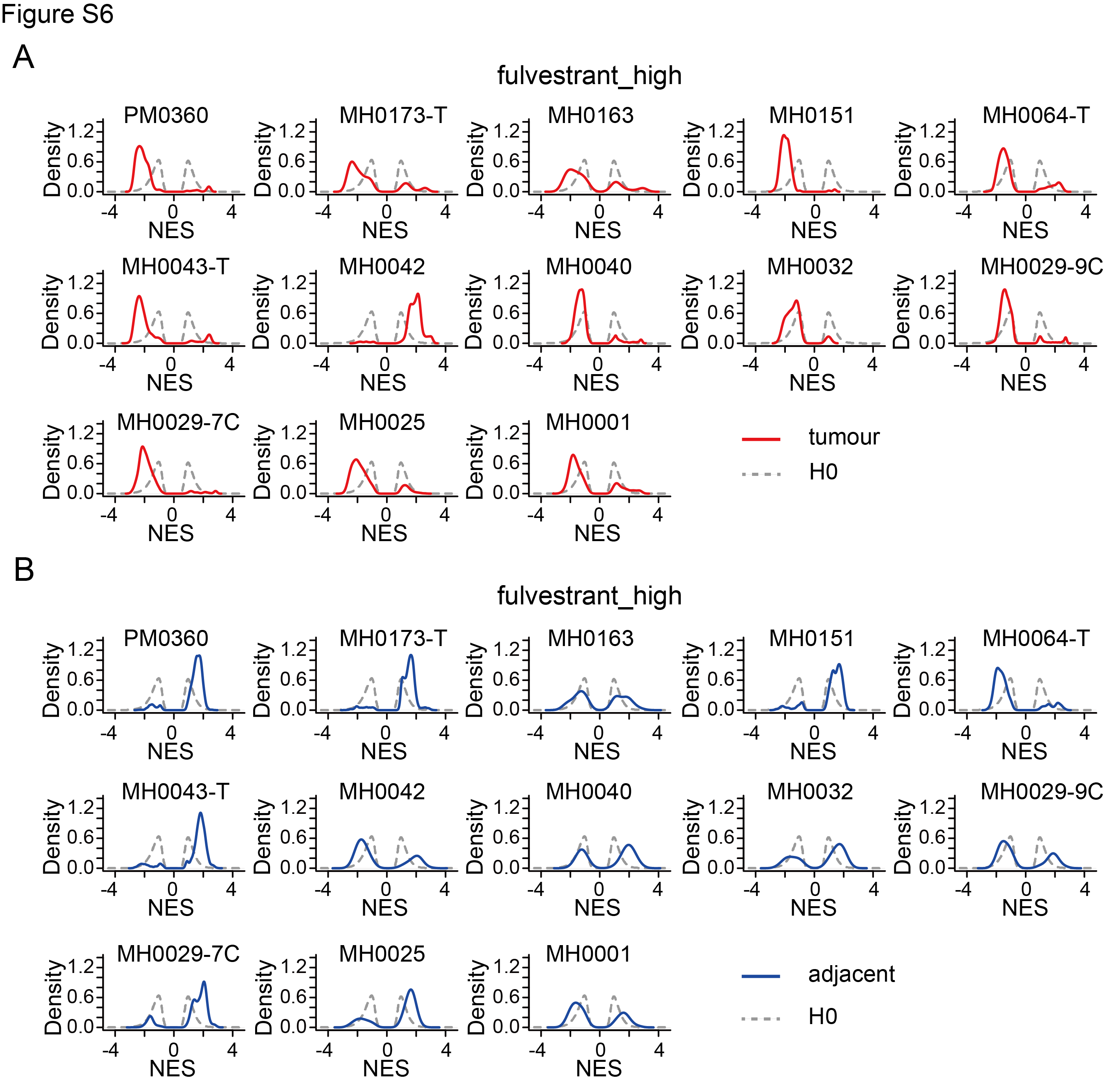
**

**Figure S6. Application of scPharm to ER-positive breast cancer in the context of fulvestrant.** **A.** Density plot depicting the NESs of single cells from 13 ER-positive breast cancer tissues (red curves) and healthy human tissues (grey curves) for fulvestrant_high. **B.** Density plot depicting the NESs of single cells from tumour-adjacent tissues and healthy human tissues for fulvestrant_high.

**
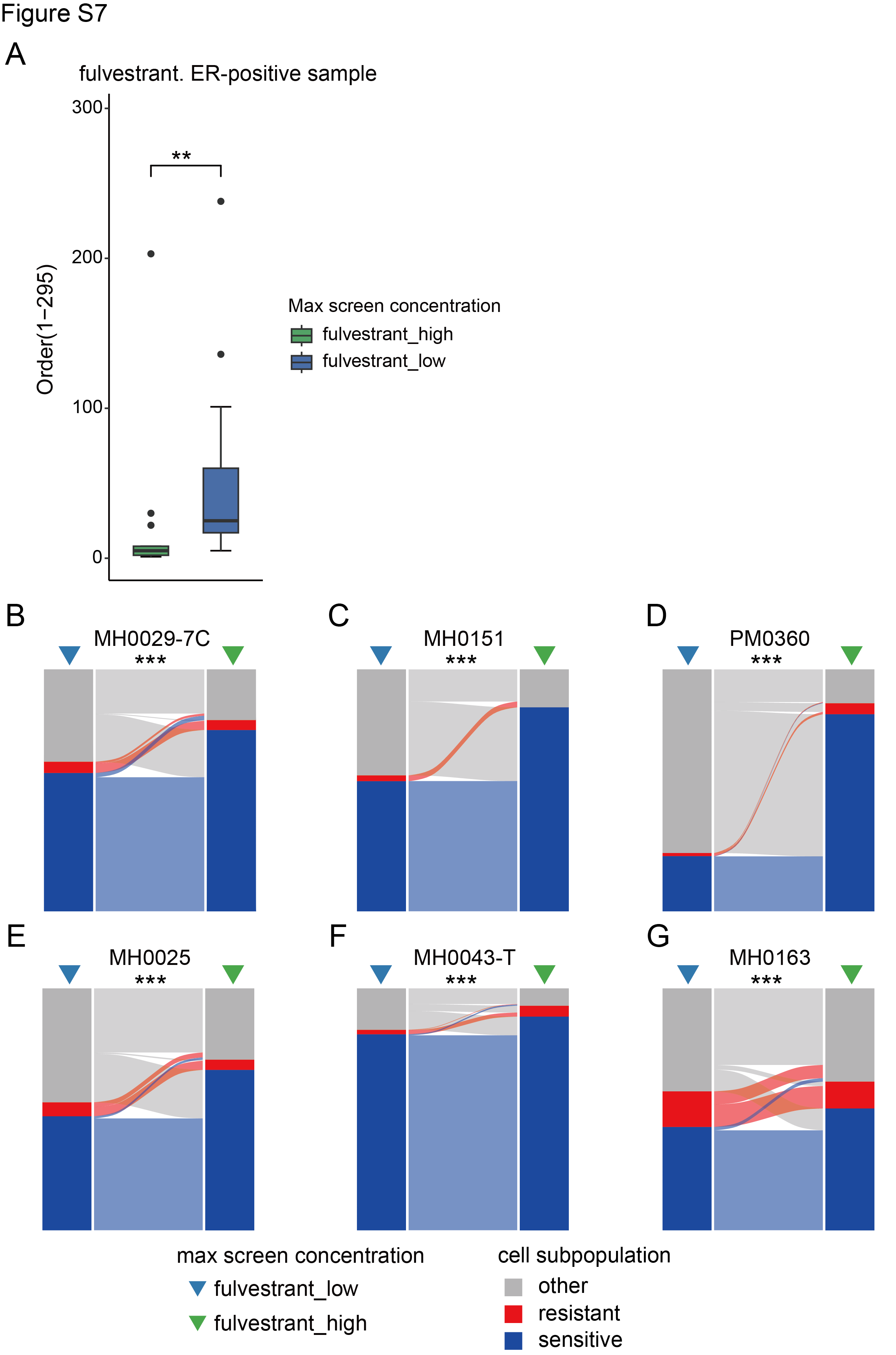
**

**Figure S7. Differences at the different maximum tested concentrations. A.** Comparative ranking of predictions for fulvestrant at various concentrations. **B-G.** Alluvial plot showing the changes in three types of subpopulations between the different concentrations across the samples. A one-sided Mann‒Whitney test was employed, with “*” indicating a *P* value less than 0.05, “**” indicating a *P* value less than 0.01, and “***” indicating a *P* value less than 0.001.

**
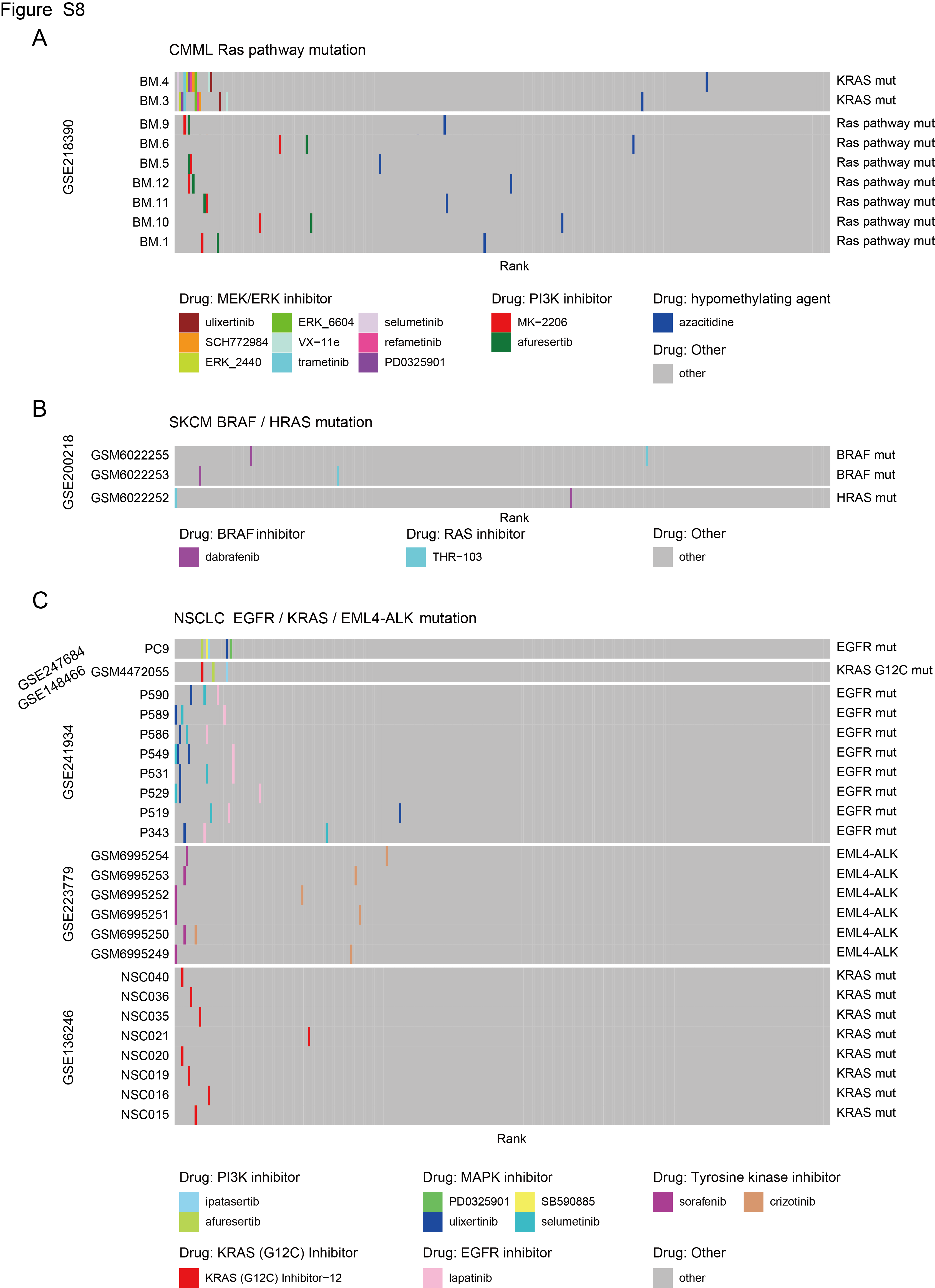
**

**Figure S8. Validation of the performance of scPharm across additional datasets.** **A.** Ranking of ERK/MEK inhibitors and PI3K inhibitors within the CMML dataset (GSE218390). Specifically, samples BM.3 and BM.4 harbour *KRAS* gene mutations, whereas other samples contain mutations in various genes within the RAS pathway. **B.** Ranking of BRAF inhibitors and RAS inhibitors within the SKCM dataset (GSE200218), which includes samples with mutations in the *BRAF* or *HRAS* genes. **C.** Ranking of targeted therapeutic drugs across various mutations (*EGFR* mutation, *KRAS* mutation, and *EML4–ALK* fusion) within the LUAD datasets (GSE247684, GSE148466, GSE241934, GSE223779, and GSE136246).

**
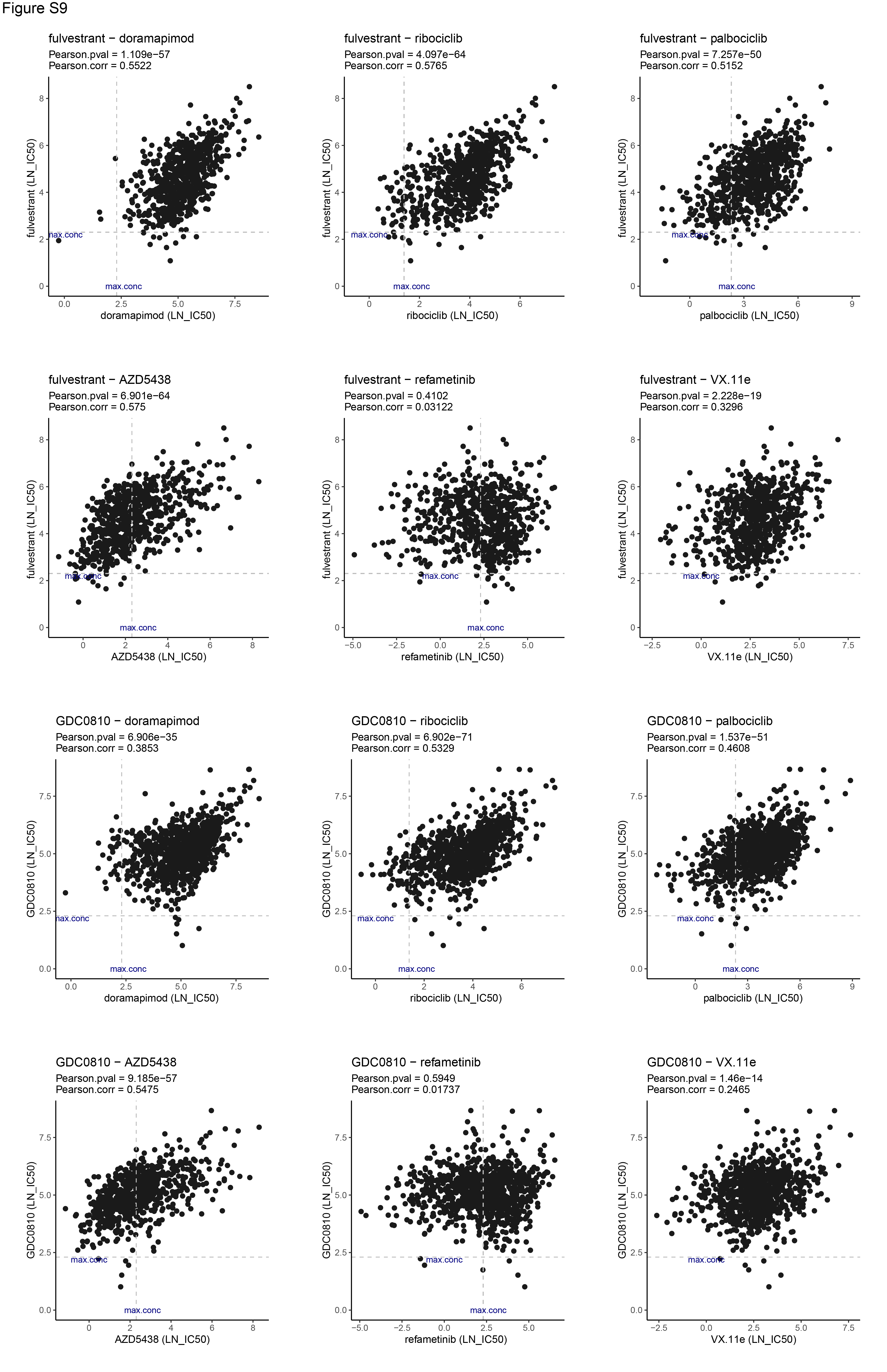
**

**Figure S9. Scatterplot of the Pearson correlation coefficients of the IC50 values for the 12 drug combinations in all the cancer cell lines**. The dashed line indicates the maximum screening concentration of the drug.

**
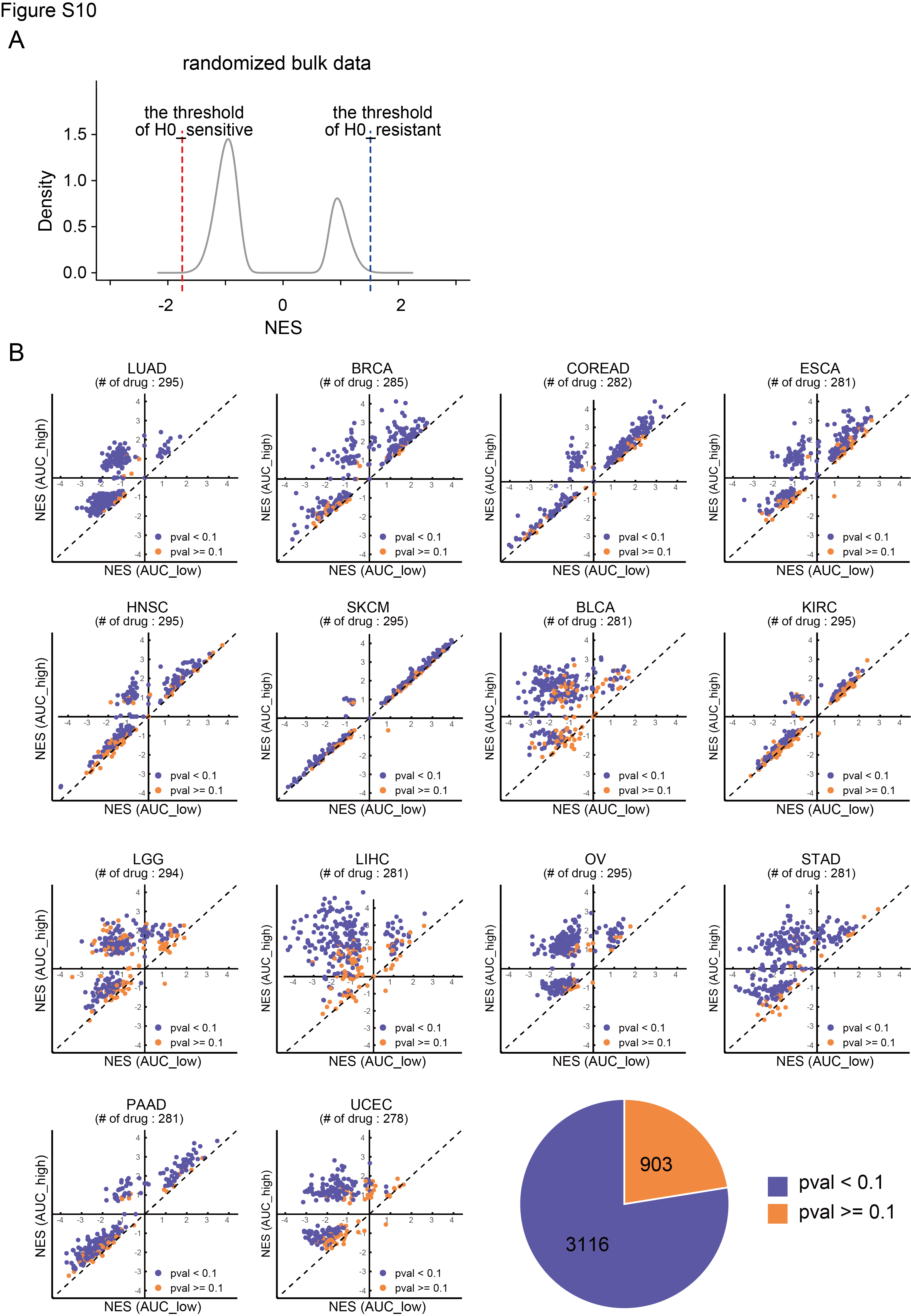
**

**Figure S10.** **The evaluation of false-positive associations of scPharm and correlation between the NES and drug response at bulk data**. **A**. Both the cell lines and gene expression profiles were shuffled to simulate a scenario with no real biological associations. The distribution of NESs when randomized bulk data used. The red and blue dashed line represents the thresholds of *H0* distributions for sensitive cells and resistant cell used in scPharm. **B**. The scatter plot shows the NESs in the AUC-high group versus the AUC-low group. The cell lines were divided into AUC-high and AUC-low groups based on the median AUC for each drug.

**Table S1 The data extracted from Mohr's, Yoldi's, and Formisano's studies**

**Mohr's study (related to Figure 4H)**

| **concentration[nM]** | **relative Ki-67 index** | **se.max^a)^** | **se** |
| --- | --- | --- | --- |
| 0.00 | 1.00 | 1.14 | 0.14 |
| 1.00 | 0.69 | 0.73 | 0.04 |
| 10.00 | 0.65 | 0.72 | 0.06 |
| 100.00 | 0.36 | 0.40 | 0.04 |

^a)^ se means standard error.

**Mohr's study (related to Figure 4H)**

| **concentration[nM]** | **relative proliferation rate** | **se.max^a)^** | **se** |
| --- | --- | --- | --- |
| 0.00 | 1.00 | 1.07 | 0.07 |
| 100.00 | 0.21 | 0.24 | 0.03 |

^a)^ se means standard error.

**Yoldi's study (related to Figure 4I)**

| **days** | **group** | **tumor volume** | **se.max^a)^** | **se** |
| --- | --- | --- | --- | --- |
| 1 | WT | 0.107493 | 0.110346 | 0.002853 |
| 3 | WT | 0.253839 | 0.257724 | 0.003885 |
| 7 | WT | 0.334135 | 0.354857 | 0.020722 |
| 10 | WT | 0.586679 | 0.644958 | 0.058279 |
| 14 | WT | 0.735615 | 0.791304 | 0.055689 |
| 21 | WT | 0.907863 | 1.03349 | 0.125627 |
| 1 | WT+docetaxel | 0.117854 | 0.11469 | 0.003164 |
| 3 | WT+docetaxel | 0.221462 | 0.237003 | 0.015541 |
| 7 | WT+docetaxel | 0.419611 | 0.425694 | 0.006083 |
| 10 | WT+docetaxel | 0.573728 | 0.600925 | 0.027197 |
| 14 | WT+docetaxel | 0.677336 | 0.672155 | 0.005181 |
| 17 | WT+docetaxel | 0.542646 | 0.542646 | 0 |
| 21 | WT+docetaxel | 0.422202 | 0.415726 | 0.006476 |
| 24 | WT+docetaxel | 0.334135 | 0.328955 | 0.00518 |
| 28 | WT+docetaxel | 0.279741 | 0.282331 | 0.00259 |
| 31 | WT+docetaxel | 0.32766 | 0.413136 | 0.085476 |
| 35 | WT+docetaxel | 0.357447 | 0.451989 | 0.094542 |
| 38 | WT+docetaxel | 0.415726 | 0.529695 | 0.113969 |

^a)^ se means standard error.

**Formisano's study (related to Figure 6D)**

| **treatment**  **[days]** | **group** | | **tumor volume [log2]** | | **se.max^a)^** | **se** |
| --- | --- | --- | --- | --- | --- | --- |
| 0 | | Vehicle | | 239.855 | 259.146 | 19.291 |
| 4 | | Vehicle | | 250.841 | 277.718 | 26.877 |
| 7 | | Vehicle | | 269.366 | 292.812 | 23.446 |
| 10 | | Vehicle | | 289.258 | 314.436 | 25.178 |
| 14 | | Vehicle | | 296.411 | 322.868 | 26.457 |
| 18 | | Vehicle | | 361.854 | 383.079 | 21.225 |
| 21 | | Vehicle | | 366.301 | 391.754 | 25.453 |
| 24 | | Vehicle | | 382.3 | 408.864 | 26.564 |
| 28 | | Vehicle | | 424.988 | 463.865 | 38.877 |
| 31 | | Vehicle | | 428.462 | 466.707 | 38.245 |
| 34 | | Vehicle | | 454.518 | 514.612 | 60.094 |
| 0 | | Fulvestrant | | 243.298 | 263.938 | 20.64 |
| 4 | | Fulvestrant | | 230.286 | 259.674 | 29.388 |
| 7 | | Fulvestrant | | 212.278 | 240.834 | 28.556 |
| 10 | | Fulvestrant | | 188.637 | 213.578 | 24.941 |
| 14 | | Fulvestrant | | 206.313 | 235.501 | 29.188 |
| 18 | | Fulvestrant | | 207.577 | 233.591 | 26.014 |
| 21 | | Fulvestrant | | 186.347 | 206.313 | 19.966 |
| 24 | | Fulvestrant | | 186.727 | 206.734 | 20.007 |
| 28 | | Fulvestrant | | 160.942 | 178.549 | 17.607 |
| 31 | | Fulvestrant | | 158.988 | 176.381 | 17.393 |
| 34 | | Fulvestrant | | 153.893 | 169 | 15.107 |
| 0 | | Fulvestrant+Palbociclib | | 254.441 | 277.153 | 22.712 |
| 4 | | Fulvestrant+Palbociclib | | 191.345 | 193.302 | 1.957 |
| 7 | | Fulvestrant+Palbociclib | | 144.482 | 145.96 | 1.478 |
| 10 | | Fulvestrant+Palbociclib | | 137.033 | 149.569 | 12.536 |
| 14 | | Fulvestrant+Palbociclib | | 150.179 | 161.928 | 11.749 |
| 18 | | Fulvestrant+Palbociclib | | 121.525 | 133.455 | 11.93 |
| 21 | | Fulvestrant+Palbociclib | | 121.525 | 132.372 | 10.847 |
| 24 | | Fulvestrant+Palbociclib | | 93.4586 | 107.991 | 14.5324 |
| 28 | | Fulvestrant+Palbociclib | | 76.4003 | 86.3257 | 9.9254 |
| 31 | | Fulvestrant+Palbociclib | | 72.4618 | 81.8755 | 9.4137 |
| 34 | | Fulvestrant+Palbociclib | | 67.0678 | 74.8608 | 7.793 |

^a)^ se means standard error.

**Table S2 The scRNA-seq datasets used for evaluating scPharm performance**

| **Datasets** | **Sample** | **ID used** | **Tissue** | **Cancer type** | **#cells used** | **#genes used** | **Genotype** | **Note** |
| --- | --- | --- | --- | --- | --- | --- | --- | --- |
| GSE161529 | GSM4909289 | AH0308 | Mammary gland | breast cancer | 4,632 | 19,911 | HER2+ | maxGene:7,000; maxUMI:40,000 |
| GSE161529 | GSM4909291 | MH0031 | Mammary gland | breast cancer | 4,593 | 18,355 | HER2+ | maxGene:3,000; maxUMI:10,000 |
| GSE161529 | GSM4909292 | MH0069 | Mammary gland | breast cancer | 561 | 14,237 | HER2+ | maxGene:4,000; maxUMI:10,000 |
| GSE161529 | GSM4909293 | MH0161 | Mammary gland | breast cancer | 6,399 | 20,004 | HER2+ | maxGene:5,000; maxUMI:20,000 |
| GSE161529 | GSM4909294 | MH0176 | Mammary gland | breast cancer | 17,267 | 22,220 | HER2+ | maxGene:4,000; maxUMI:10,000 |
| GSE161529 | GSM4909290 | PM0337 | Mammary gland | breast cancer | 11,309 | 19,771 | HER2+ | maxGene:5,500; maxUMI:25,000 |
| GSE161529 | GSM4909296 | MH0001 | Mammary gland | breast cancer | 7,092 | 16,560 | ER+ | maxGene:1,500; maxUMI:5,000 |
| GSE161529 | GSM4909302 | MH0025 | Mammary gland | breast cancer | 9,518 | 20,064 | ER+ | maxGene:2,500; maxUMI:7,500 |
| GSE161529 | GSM4909305 | MH0029.7C | Mammary gland | breast cancer | 3,114 | 18,980 | ER+ | maxGene:4,000; maxUMI:15,000 |
| GSE161529 | GSM4909306 | MH0029.9C | Mammary gland | breast cancer | 7,647 | 19,486 | ER+ | maxGene:3,500; maxUMI:15,000 |
| GSE161529 | GSM4909300 | MH0032 | Mammary gland | breast cancer | 1,278 | 17,171 | ER+ | maxGene:3,000; maxUMI:10,000 |
| GSE161529 | GSM4909307 | MH0040 | Mammary gland | breast cancer | 6,006 | 17,701 | ER+ | maxGene:2,500; maxUMI:10,000 |
| GSE161529 | GSM4909301 | MH0042 | Mammary gland | breast cancer | 4,884 | 17,411 | ER+ | maxGene:2,500; maxUMI:5,000 |
| GSE161529 | GSM4909309 | MH0043.T | Mammary gland | breast cancer | 4,970 | 18,624 | ER+ | maxGene:2,500; maxUMI:7,000 |
| GSE161529 | GSM4909313 | MH0064.T | Mammary | breast cancer | 3,703 | 17,040 | ER+ | maxGene:2,000; maxUMI:5,000 |
| GSE161529 | GSM4909303 | MH0151 | Mammary gland | breast cancer | 2,483 | 20,393 | ER+ | maxGene:4,000; maxUMI:7,500 |
| GSE161529 | GSM4909304 | MH0163 | Mammary gland | breast cancer | 7,159 | 18,687 | ER+ | maxGene:3,500; maxUMI:12,000 |
| GSE161529 | GSM4909317 | MH0173.T | Mammary gland | breast cancer | 8,730 | 21,505 | ER+ | maxGene:4,000; maxUMI:12,000 |
| GSE161529 | GSM4909298 | PM0360 | Mammary gland | breast cancer | 5,725 | 19,177 | ER+ | maxGene:2,500; maxUMI:7,500 |
| GSE158677 | GSM4805459 | T1 | Mammary | breast cancer | 492 | 18,928 | MMTV-PyMT | Epithelial cells only, identified by gene marker ("PyMT","Epcam","Krt8") |
| GSE158677 | GSM4805460 | T2 | Mammary | breast cancer | 405 | 18,296 | MMTV-PyMT | Epithelial cells only, identified by gene marker ("PyMT","Epcam","Krt8") |
| GSE158677 | GSM4805461 | T3 | Mammary | breast cancer | 1,169 | 20,977 | MMTV-PyMT | Epithelial cells only, identified by gene marker ("PyMT","Epcam","Krt8") |
| GSE158677 | GSM4805462 | T4 | Mammary | breast cancer | 2,940 | 19,555 | MMTV-PyMT | Epithelial cells only, identified by gene marker ("PyMT","Epcam","Krt8") |
| GSE158677 | GSM4805463 | T5 | Mammary | breast cancer | 1,438 | 22,314 | MMTV-PyMT | Epithelial cells only, identified by gene marker ("PyMT","Epcam","Krt8") |
| GSE134839 | GSM3972658 | Day 1 | PC9 | lung adenocarcinomas | 242 | 18,461 | EGFR-mutant | maxGene:7,500; minGene:800 |
| GSE134839 | GSM3972659 | Day 2 | PC9 | lung adenocarcinomas | 217 | 18,461 | EGFR-mutant | maxGene:7,500; minGene:800 |
| GSE134839 | GSM3972660 | Day 4 | PC9 | lung adenocarcinomas | 199 | 18,461 | EGFR-mutant | maxGene:7,500; minGene:800 |
| GSE134839 | GSM3972661 | Day 9 | PC9 | lung adenocarcinomas | 387 | 18,461 | EGFR-mutant | maxGene:7,500; minGene:800 |
| GSE134839 | GSM3972662 | Day 11 | PC9 | lung adenocarcinomas | 266 | 18,461 | EGFR-mutant | maxGene:7,500; minGene:800 |
| GSE136246 | GSM4043245 | NSC015 | Lung | lung adenocarcinomas | 4,383 | 31,614 | KRAS-mutant | maxGene:5,000; maxUMI:12,000 |
| GSE136246 | GSM4043246 | NSC015 | Lung | lung adenocarcinomas |  |  | KRAS-mutant | maxGene:5,000; maxUMI:12,000 |
| GSE136246 | GSM4043247 | NSC015 | Lung | lung adenocarcinomas |  |  | KRAS-mutant | maxGene:5,000; maxUMI:12,000 |
| GSE136246 | GSM4043248 | NSC016 | Lung | lung adenocarcinomas | 8,967 | 35,051 | KRAS-mutant | maxGene:5,000; maxUMI:12,000 |
| GSE136246 | GSM4043249 | NSC016 | Lung | lung adenocarcinomas |  |  | KRAS-mutant | maxGene:5,000; maxUMI:12,000 |
| GSE136246 | GSM4043250 | NSC016 | Lung | lung adenocarcinomas |  |  | KRAS-mutant | maxGene:5,000; maxUMI:12,000 |
| GSE136246 | GSM4043251 | NSC019 | Lung | lung adenocarcinomas | 3,851 | 32,353 | KRAS-mutant | maxGene:5,000; maxUMI:12,000 |
| GSE136246 | GSM4043252 | NSC019 | Lung | lung adenocarcinomas |  |  | KRAS-mutant | maxGene:5,000; maxUMI:12,000 |
| GSE136246 | GSM4043253 | NSC020 | Lung | lung adenocarcinomas | 3,370 | 28,970 | KRAS-mutant | maxGene:5,000; maxUMI:12,000 |
| GSE136246 | GSM4043254 | NSC020 | Lung | lung adenocarcinomas |  |  | KRAS-mutant | maxGene:5,000; maxUMI:12,000 |
| GSE136246 | GSM4043255 | NSC021 | Lung | lung adenocarcinomas | 5,505 | 33,532 | KRAS-mutant | maxGene:5,000; maxUMI:12,000 |
| GSE136246 | GSM4043256 | NSC021 | Lung | lung adenocarcinomas |  |  | KRAS-mutant | maxGene:5,000; maxUMI:12,000 |
| GSE136246 | GSM4043257 | NSC035 | Lung | lung adenocarcinomas | 1,150 | 21,644 | KRAS-mutant | maxGene:5,000; maxUMI:12,000 |
| GSE136246 | GSM4043258 | NSC036 | Lung | lung adenocarcinomas | 1,025 | 19,605 | KRAS-mutant | maxGene:5,000; maxUMI:12,000 |
| GSE136246 | GSM4043260 | NSC040 | Lung | lung adenocarcinomas | 914 | 22,684 | KRAS-mutant | maxGene:5,000; maxUMI:12,000 |
| GSE218390 | GSM6744114 | BM.10 | Bone Marrow | chronic myelomonocytic leukemia (CMML) | 1,398 | 18,305 | genotype: PTPN11 A72T | RAS pathway mutant |
| GSE218390 | GSM6744115 | BM.11 | Bone Marrow | chronic myelomonocytic leukemia (CMML) | 327 | 16,026 | genotype: BRAF G469A, CBL K382E, CBL C384Y | RAS pathway mutant |
| GSE218390 | GSM6744116 | BM.12 | Bone Marrow | chronic myelomonocytic leukemia (CMML) | 544 | 17,091 | genotype: BRAF G469A | RAS pathway mutant |
| GSE218390 | GSM6744117 | BM.1 | Bone Marrow | chronic myelomonocytic leukemia (CMML) | 4,210 | 19,688 | genotype: CBL Y371H, NRAS Y64_S6, NRAS G13V, NRAS G12S | RAS pathway mutant |
| GSE218390 | GSM6744119 | BM.3 | Bone Marrow | chronic myelomonocytic leukemia (CMML) | 500 | 17,623 | genotype: KRAST58I, NRASG12R, KRAS T58K | RAS pathway mutant |
| GSE218390 | GSM6744120 | BM.4 | Bone Marrow | chronic myelomonocytic leukemia (CMML) | 560 | 18,088 | genotype: KRAS T58I, NRAS G12R, KRAS T58K | RAS pathway mutant |
| GSE218390 | GSM6744121 | BM.5 | Bone Marrow | chronic myelomonocytic leukemia (CMML) | 2,873 | 19,027 | genotype: CBL C384Y | RAS pathway mutant |
| GSE218390 | GSM6744122 | BM.6 | Bone Marrow | chronic myelomonocytic leukemia (CMML) | 3,160 | 19,189 | genotype: CBL C384Y, CBL F378fs | RAS pathway mutant |
| GSE218390 | GSM6744125 | BM.9 | Bone Marrow | chronic myelomonocytic leukemia (CMML) | 662 | 16,842 | genotype: PTPN11 A72T | RAS pathway mutant |
| GSE200218 | GSM6022252 | GSM6022252 | Melanoma brain metastases | Melanoma | 3,280 | 36,601 | genotype: HRAS T58I |  |
| GSE200218 | GSM6022253 | GSM6022253 | Melanoma brain metastases | Melanoma | 4,814 | 36,601 | genotype: BRAF V600E |  |
| GSE200218 | GSM6022255 | GSM6022255 | Melanoma brain metastases | Melanoma | 3,430 | 36,601 | genotype: BRAF(R178*, c.532C>T) |  |
| GSE247684 | GSM7898876 | PC9 | PC9 | lung adenocarcinoma | 11,258 | 15,429 | genotype: EGFR-del |  |
| GSE247684 | GSM7898877 | PC9 | PC9 | lung adenocarcinoma |  |  | genotype: EGFR-del |  |
| GSE148466 | GSM4472055 | GSM4472055 | Lung | lung squamous cell carcinoma | 11,983 | 24,374 | genotype: KRAS p.G12C | Adenocarcinoma with focal squamous differentiation |
| GSE241934 | GSM7745818 | P343 | Lung | lung adenocarcinoma | 7,051 | 27,693 | EGFR-mutant |  |
| GSE241934 | GSM7745820 | P519 | Lung | lung adenocarcinoma | 6,649 | 27,693 | EGFR-mutant |  |
| GSE241934 | GSM7745821 | P529 | Lung | lung adenocarcinoma | 7,275 | 27,693 | EGFR-mutant |  |
| GSE241934 | GSM7745822 | P531 | Lung | lung adenocarcinoma | 7,459 | 27,693 | EGFR-mutant |  |
| GSE241934 | GSM7745824 | P549 | Lung | lung adenocarcinoma | 10,295 | 27,693 | EGFR-mutant |  |
| GSE241934 | GSM7745825 | P586 | Lung | lung adenocarcinoma | 6,822 | 27,693 | EGFR-mutant |  |
| GSE241934 | GSM7745826 | P589 | Lung | lung adenocarcinoma | 6,689 | 27,693 | EGFR-mutant |  |
| GSE241934 | GSM7745827 | P590 | Lung | lung adenocarcinoma | 7,792 | 27,693 | EGFR-mutant |  |
| GSE223779 | GSM6995249 | GSM6995249 | Lung | lung adenocarcinoma | 9,456 | 33,538 | genotype: EML4-ALK |  |
| GSE223779 | GSM6995250 | GSM6995250 | Lung | lung adenocarcinoma | 5,934 | 33,538 | genotype: EML4-ALK |  |
| GSE223779 | GSM6995251 | GSM6995251 | Lung | lung adenocarcinoma | 8,282 | 33,538 | genotype: EML4-ALK |  |
| GSE223779 | GSM6995252 | GSM6995252 | Lung | lung adenocarcinoma | 11,153 | 33,538 | genotype: EML4-ALK |  |
| GSE223779 | GSM6995253 | GSM6995253 | Lung | lung adenocarcinoma | 11,981 | 33,538 | genotype: EML4-ALK |  |
| GSE223779 | GSM6995254 | GSM6995254 | Lung | lung adenocarcinoma | 10,030 | 33,538 | genotype: EML4-ALK |  |

**Table S3 The scRNA-seq datasets used for modelling the null distribution of NES**

| **Datasets** | **Sample** | **Tissue** | **#cells used** | **#genes used** |
| --- | --- | --- | --- | --- |
| GSE196638 | GSM5897133 | Healthy Lung | 12,247 | 24,013 |
| GSE196638 | GSM5897134 | Healthy Lung | 7,218 | 24,013 |
| GSE196638 | GSM5897135 | Healthy Lung | 8,322 | 24,013 |
| GSE150247 | GSM4544005 | Healthy Lung | 5,482 | 24,013 |
| GSE150247 | GSM4544006 | Healthy Lung | 8,433 | 24,013 |
| GSE150247 | GSM4544007 | Healthy Lung | 8,226 | 24,013 |
| GSE122960 | GSM3489182 | Healthy Lung | 7,109 | 24,013 |
| GSE122960 | GSM3489185 | Healthy Lung | 6,744 | 24,013 |
| GSE122960 | GSM3489187 | Healthy Lung | 7,008 | 24,013 |
| GSE122960 | GSM3489189 | Healthy Lung | 9,273 | 24,013 |
| GSE122960 | GSM3489191 | Healthy Lung | 9,837 | 24,013 |
| GSE122960 | GSM3489193 | Healthy Lung | 9,080 | 24,013 |
| GSE122960 | GSM3489195 | Healthy Lung | 14,111 | 24,013 |
| GSE164898 | GSM5022599 | Healthy Mammary gland | 4,573 | 22,999 |
| GSE164898 | GSM5022600 | Healthy Mammary gland | 1,145 | 22,999 |
| GSE164898 | GSM5022601 | Healthy Mammary gland | 4,631 | 22,999 |
| GSE164898 | GSM5022602 | Healthy Mammary gland | 3,120 | 22,999 |
| GSE164898 | GSM5022603 | Healthy Mammary gland | 4,301 | 22,999 |
| GSE164898 | GSM5022604 | Healthy Mammary gland | 10,249 | 22,999 |
| GSE164898 | GSM5022605 | Healthy Mammary gland | 9,156 | 22,999 |
| GSE164898 | GSM5022606 | Healthy Mammary gland | 7,533 | 22,999 |
| GSE151177 | GSM4567877 | Normal Skin | 2,483 | 18,133 |
| GSE151177 | GSM4567878 | Normal Skin | 957 | 18,133 |
| GSE151177 | GSM4567879 | Normal Skin | 343 | 18,133 |
| GSE151177 | GSM4567880 | Normal Skin | 1,501 | 18,133 |
| GSE151177 | GSM4567881 | Normal Skin | 101 | 18,133 |

**Data availability**

The scRNA-seq datasets analysed during the current study are available in the Gene Expression Omnibus (GEO) [https://www.ncbi.nlm.nih.gov/geo/] and Single Cell Portal (SCP) [https://singlecell.broadinstitute.org/single_cell]. For detailed information on the scRNA-seq datasets used for the evaluation, refer to Table S2; For information on the method used to generate the null distribution of NESs, refer to Table S3. All processed data (gene expression profiles) are available in Zenodo [https://doi.org/10.5281/zenodo.13690742]. Pharmacologic data were downloaded from the GDSC database [https://www.cancerrxgene.org/downloads/]. The data from the studies of Mohr, Yoldi, and Formisano used in our study are organized in Table S1.

**Code availability**

scPharm is implemented in R and is freely available from GitHub [https://github.com/WangHYLab/scPharm]. The analysis and plotting code are also available on GitHub [https://github.com/WangHYLab/scPharm_ms].
